# Vinegar Consumption and Health: An Umbrella Review of Meta‐Analyses of Randomized Controlled Trials

**DOI:** 10.1002/fsn3.71849

**Published:** 2026-05-06

**Authors:** Forough Shahmohammadi, Reza Amiri Khosroshahi, Hoda Derakhshanian, Hamed Mohammadi

**Affiliations:** ^1^ Department of Clinical Nutrition, School of Nutritional Sciences and Dietetics Tehran University of Medical Sciences Tehran Iran; ^2^ Students' Scientific Research Center (SSRC) Tehran University of Medical Sciences Tehran Iran; ^3^ Student Research Committee Tabriz University of Medical Sciences Tabriz Iran; ^4^ Department of Cellular and Molecular Nutrition, School of Nutritional Sciences and Dietetics Tehran University of Medical Sciences Tehran Iran

**Keywords:** anthropometric indicators, blood pressure, glycemic control, lipid profile, meta‐analysis, vinegar

## Abstract

Vinegar is a natural dietary product that has been proposed to exert beneficial effects on cardiometabolic health, including glycemic regulation, lipid metabolism, blood pressure control, and weight management. Although several meta‐analyses have reported favorable outcomes associated with vinegar consumption, the overall strength and quality of the available evidence have not been comprehensively evaluated. We systematically searched PubMed, Scopus, Web of Science, and Google Scholar for relevant studies published up to January 2025. Effect sizes from eligible meta‐analyses were re‐estimated using a random‐effects model. Ten meta‐analyses, comprising a total of 38 primary studies, were included. Pooled results indicated that vinegar consumption was associated with improvements in fasting blood glucose (FBG) (−9.40 mg/dL), postprandial glucose (PPG) (−14.59 mg/dL), postprandial insulin (PPI) (−1.29 mu/L), hemoglobin A1c (HbA1c) (−0.70%), total cholesterol (TC) (−9.39 mg/dL), body weight (−1.06 kg), and systolic blood pressure (SBP) (−2.94 mmHg). In contrast, no significant effects were observed for fasting plasma insulin (FPI), insulin resistance assessed by homeostatic model assessment of insulin resistance (HOMA‐IR), low‐density lipoprotein (LDL), and high‐density lipoprotein (HDL), triglycerides (TG), body mass index (BMI), waist circumference (WC), or diastolic blood pressure (DBP). Vinegar intake was associated with statistically significant improvements in FBG (moderate certainty), PPG (moderate certainty), HbA1c (moderate certainty), TC (very low certainty), body weight (high certainty), and SBP (high certainty). No significant effects were observed for other outcomes. The certainty of evidence ranged from very low to high across outcomes.

## Graphical abstract

Vinegar consumption, particularly apple cider vinegar, may improve glycemic control, lipid profile, body weight, and blood pressure through multiple mechanisms. These effects are partly mediated by delayed gastric emptying, modulation of glucose metabolism, enhanced fatty acid oxidation, reduced lipogenesis, and increased satiety. Overall, vinegar intake shows significant benefits on several metabolic parameters, although some outcomes remain non‐significant.

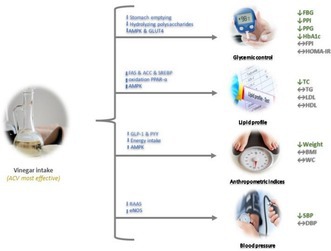

**Abbreviations**
AAB: acetic acid bacteria
ACV: apple cider vinegar
AMPK: adenosine monophosphate‐activated protein kinase
AMSTAR: A Measurement Tool to Assess Systematic Reviews
BMI: body mass index
CI: confidence intervals
DBP: diastolic blood pressure
FBG: fasting blood glucose
FPI: fasting plasma insulin
GLP‐1: glucagon‐like peptide‐1
GLUT4: Glucose transporter type 4
GRADE: Grading of Recommendations Assessment Development and Evaluation
HbA1c: hemoglobin A1c
HDL: high‐density lipoprotein
HOMA‐IR: homeostatic model assessment of insulin resistance
LDL: low‐density lipoprotein
MD: mean differences
PEPCK: phosphoenolpyruvate carboxy‐kinase
PPG: post prandial glucose
PPI: post prandial insulin
PRIOR: Preferred Reporting Items for Overviews of Reviews
PROSPERO: International Prospective Register of Systematic Reviews
PYY: peptide YY
RAAS: renin‐angiotensin‐aldosterone system
RCT: randomized controlled trial
RoB: risk of bias
SBP: systolic blood pressure
SRMA: systematic reviews and meta‐analyses
T2DM: type two diabetes mellitus
TC: total cholesterol
TG: triglycerides
WC: waist circumference

## 1 Introduction

Vinegar is a naturally occurring food, primarily composed of acetic acid and bioactive compounds such as polyphenols, melanoidins, and organic acids (Chen et al. 2016; Samad et al. 2016). It is produced through a two‐step fermentation process, whereby yeasts first convert sugars into alcohol, which is then oxidized to acetic acid by acetic acid bacteria (AAB) (Gullo and Giudici 2008). Vinegar can be classified as grain‐based (e.g., sorghum, rice, wheat) or fruit‐based (e.g., grapes, apples) (Solieri and Giudici 2009). Historically, it has been employed medicinally to alleviate gastrointestinal and metabolic conditions, and it remains a widely used preservative and flavoring agent in foods such as pickles, mayonnaise, ketchup, and salad dressings (Bray 2014; Saha and Banerjee 2013).

Recent studies have highlighted additional potential benefits, including antibacterial, antioxidative, anti‐cancer, glycemic‐regulating, lipid‐modulating, blood pressure‐lowering, cardiovascular‐protective, and weight‐management effects (Budak et al. 2014; Chen et al. 2016). Research has examined these effects across diverse conditions, including diabetes (Johnston et al. 2004; Leeman et al. 2005), cancer (Nanda et al. 2004), cardiovascular disease (Beheshti et al. 2012; Mahdavi‐Roshan et al. 2022), gastrointestinal disorders (Johnston 2009), and obesity (Abou‐Khalil et al. 2024).

Nevertheless, evidence regarding vinegar's influence on glycemic control, lipid profiles, anthropometric indicators, and blood pressure is inconsistent (Fakhri et al. 2022; Keshani et al. 2025; Sohouli et al. 2022). Several meta‐analyses have reported significant improvements in FBG (Arjmandfard et al. 2025; Siddiqui et al. 2018), however, one meta‐analysis found no significant impact on healthy, overweight, or obese patients and metabolic conditions (Valdes et al. 2021). The results of certain meta‐analyses on vinegar consumption point to significant changes in HbA1C (Arjmandfard et al. 2025; Siddiqui et al. 2018), TG (Hadi et al. 2021), LDL (Cheng et al. 2020; Sohouli et al. 2022), BMI (Sohouli et al. 2022), SBP (Shahinfar et al. 2022), and DBP (Shahinfar et al. 2022). Nevertheless, conflicting evidence suggests that there are no significant differences in HbA1C (Sohouli et al. 2022; Valdes et al. 2021), TG (Cheng et al. 2020; Sohouli et al. 2022), LDL (Hadi et al. 2021; Keshani et al. 2025), BMI (Keshani et al. 2025; Valdes et al. 2021), SBP (Fakhri et al. 2022), and DBP (Fakhri et al. 2022).

Although systematic reviews and meta‐analyses (SRMAs) have explored the health effects of vinegar, the type of vinegar exerting the greatest impact and the certainty of evidence supporting these effects have not been fully assessed. Accordingly, this umbrella review aims to synthesize and critically evaluate current evidence on the effects of vinegar consumption on glycemic control, lipid profile, anthropometric outcomes, and blood pressure.

## 2 Methods

The present umbrella review was carried out by the Cochrane Handbook for interpreting “overviews of reviews” (Higgins et al. 2024) and utilized the Grading of Recommendations Assessment, Development, and Evaluation (GRADE) methodology (Guyatt et al. 2008). This umbrella review was reported in accordance with the Preferred Reporting Items for Overviews of Reviews (PRIOR) checklist (Gates et al. 2022; Pollock et al. 2019) (Table S1). The registration number for this research study, as assigned by the International Prospective Register of Systematic Reviews (PROSPERO) database, is CRD42025649162.

### 2.1 Systematic Search

Up to January 2025, two authors (F.SH. and R.A.K.) conducted independent searches of the PubMed, Scopus, ISI Web of Science, and Google Scholar databases to identify meta‐analyses assessing the impact of vinegar consumption on health outcomes. The bibliographies of pertinent systematic reviews and meta‐analyses were precisely examined, and studies that satisfied the inclusion criteria were integrated accordingly. The methodology employed for the search, along with the associated keywords, is comprehensively outlined in Table S2. The PICOS criteria, which encompasses Participants, Intervention, Comparisons, Outcomes, and Study design, is presented in Table 1 for this study.

**TABLE 1:** PICOS criteria.

Parameter	Criteria
Participants	Adult participants
Intervention	Vinegar
Comparisons	Placebo or no intervention
Outcomes	FBG, PPG, HbA1C, FPI, PPI, HOMA‐IR, HDL, LDL, TC, TG, weight, BMI, WC, SBP and DBP
Study design	Systematic review and meta‐analyses of randomized controlled trials

Abbreviations: BMI, body mass index; DBP, diastolic blood pressure; FBG, fasting blood glucose; FPI, fasting plasma insulin; HbA1c, hemoglobin A1c; HDL, high‐density lipoprotein; HOMA‐IR, homeostatic model assessment of insulin resistance; LDL, low‐density lipoprotein; PICOS, population, intervention, comparison, outcome, study design; PPG, post prandial glucose; PPI, post prandial insulin; SBP, systolic blood pressure; TC, Total cholesterol; TG, Triglycerides.

### 2.2 Study Selection

This overview of SRMA encompasses studies that met the following criteria: (1) inclusion of randomized controlled trials (RCTs) involving adult participants aged 18 years and older; (2) assessment of the effects of vinegar consumption in comparison to a control group; (3) provision of weighted or standard mean differences (MD) along with 95% confidence intervals (CIs) for relevant outcomes; and (4) reporting of at least one variable associated with glycemic control (including FBG, PPG, HbA1C, FPI, PPI, and HOMA‐IR), lipid profile (such as HDL, LDL, TC, and TG), anthropometric indicators (including weight, BMI, and WC), as well as blood pressure readings (SBP and DBP). Narrative reviews, systematic reviews lacking meta‐analyses, studies that do not report effect sizes, and experimental studies were excluded. In instances where multiple meta‐analyses were available for a given outcome, the meta‐analysis that included the highest number of RCTs was selected. Furthermore, we conducted a manual examination of the reference lists from other meta‐analyses to uncover additional pertinent trials.

### 2.3 Data Extraction

Independent researchers (F.SH. and R.A.K.) extracted various data points from the involved SRMAs, which included the first author's name, publication year, country of origin, study design, participant gender, mean age of the study population, health condition of participants, type of vinegar, administered vinegar dosage, control group type, intervention duration, participant numbers in both intervention and control groups, and the types of outcomes measured (e.g., FBG, PPG, HbA1C, FPI, PPI, HOMA‐IR, HDL, LDL, TC, TG, weight, BMI, WC, SBP and DBP), as well as the effect sizes (MD and 95% CIs). Disputes arising between the two authors were resolved through discussion with a third author (H.M.).

### 2.4 Evaluation of Methodological Quality

The methodological quality of the included SRMAs was assessed using the “A Measurement Tool to Assess Systematic Reviews” (AMSTAR2) (Shea et al. 2017). Additionally, the quality of the RCTs in the meta‐analysis was evaluated with the Cochrane risk‐of‐bias tool for randomized trials (RoB) (J. P. Higgins et al. 2011) (Table S3). Independent reviewers (F.SH. and R.A.K.) conducted these quality assessments, and any conflicts were resolved through consensus with a third reviewer (H. M.).

### 2.5 Data Synthesis and Analysis

For the quantitative synthesis, we incorporated any available RCTs that provided data on a clinical outcome, regardless of the SRMAs in which they were included. The MD and 95% CIs were recalculated using a random‐effects model in each meta‐analysis, considering both within‐study and between‐study heterogeneity and study‐specific findings shown in the forest plots of each meta‐analysis (DerSimonian and Laird 1986). All outcome measures were converted to standardized units. For studies reporting medians and interquartile ranges (IQR), means and SDs were estimated using established methods (Luo et al. 2018; Wan et al. 2014). Effect sizes were calculated as MD when outcomes shared the same scale, and SMD when scales differed, ensuring consistent and reproducible comparisons across studies. Statistical evaluation of heterogeneity was performed using the *I* ^2^ statistic, and a chi‐square test was conducted to assess homogeneity at a significance level of *p*_heterogeneity_ < 0.10 (Cumpston et al. 2019). The Cochrane Handbook advises evaluating *I* ^2^ values based on the following guidelines: values of 0%–40% suggest low or no heterogeneity, values of 30%–60% indicate moderate heterogeneity; values of 50%–90% suggest substantial heterogeneity; and values of 75%–100% signify considerable heterogeneity (Cumpston et al. 2019). A subgroup analysis was carried out to determine the cause of the heterogeneity by examining factors including health conditions, the type of vinegar, the dose of vinegar, and the duration of the intervention. Assessment of publication bias involved the visual examination of funnel plots and the *p* values obtained from Egger's test (Egger et al. 1997) and Begg's test. Data analyses were conducted with STATA statistical software, version 14.0 (Stata Corp, College Station, TX, USA), and a *p*‐value < 0.05 was considered statistically significant.

### 2.6 Grading of the Evidence

The evidence was evaluated based on the GRADE method to determine the level of certainty (Guyatt et al. 2008). The GRADE framework comprises five key domains: risk of bias within individual studies, inconsistency, indirectness, imprecision, and publication bias. The certainty of evidence was evaluated and rated as high, medium, low, or very low grade. The quality of the evidence for the results presented in this umbrella review is described in Table S4. GRADE ratings were defined as follows: High certainty indicates strong confidence that the estimated effect closely reflects the true effect; Moderate certainty indicates moderate confidence, with the possibility that the true effect may differ meaningfully from the estimate; Low certainty reflects limited confidence, suggesting the true effect could differ substantially; Very low certainty denotes minimal confidence, implying that the true effect is likely markedly different from the estimate.

## 3 Results

### 3.1 Literature Search

**FIGURE 1:**
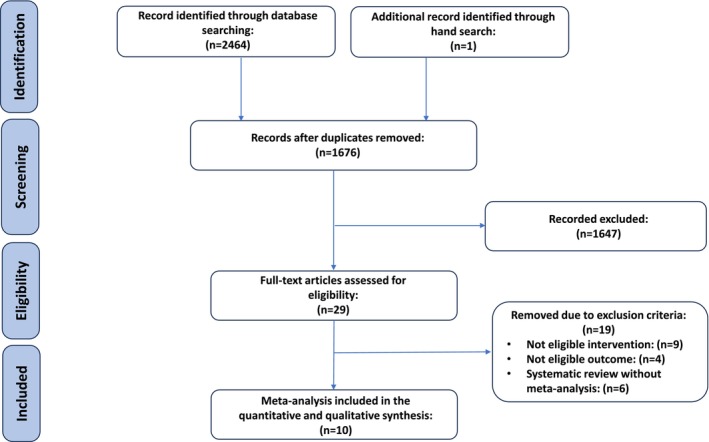
The study flow chart.

The initial search yielded 2465 pertinent results (Figure 1). After eliminating 789 duplicate studies, 1676 publications were evaluated, focusing on titles and abstracts. Among the remaining studies, 29 records were identified for a comprehensive evaluation of their full texts. Finally, this umbrella review incorporated 10 meta‐analyses (Arjmandfard et al. 2025; Cheng et al. 2020; Fakhri et al. 2022; Hadi et al. 2021; Shahinfar et al. 2022; Shishehbor et al. 2017; Siddiqui et al. 2018; Sohouli et al. 2022; Tehrani et al. 2025; Valdes et al. 2021). The Table S5 (Astbury 2024; Brown et al. 2024; Chan et al. 2024; DeSalvo et al. 2019; Feng et al. 2024; Gill et al. 2018; Harrison et al. 2023; Hasan et al. 2022; Launholt et al. 2020; Liu et al. 2024; Marmitt et al. 2021; Medina‐Vera et al. 2021; Neffe‐Skocińska et al. 2023; Sadia et al. 2018; Shahrajabian et al. 2023; T. Wang et al. 2009; Weber et al. 2023; Xavier et al. 2024; Zhang et al. 2023) presents a list of studies excluded during the full‐text assessment and provides the corresponding reasons for their exclusion.

### 3.2 Study Characteristics

A total of ten meta‐analyses, including 38 primary studies (Abid et al. 2020; Ali et al. 2018; Chiu et al. 2020; Derakhshandeh‐Rishehri et al. 2014; Ebrahimi‐Mamaghani et al. 2009; Gheflati et al. 2019; Golzarand et al. 2008; Golzarand et al. 2009; Halima et al. 2017; Hamer and Van Loon 2012; Jafarirad et al. 2023; Jasbi et al. 2019; Johnston and Buller 2005; Johnston et al. 2004; Johnston et al. 2013; Johnston et al. 2010; Johnston et al. 2009; Kahraman et al. 2011; Kausar et al. 2019; Khezri et al. 2018; Kondo, Kishi, Fushimi, Ugajin, and Kaga 2009; Liatis et al. 2010; Mahmoodi et al. 2013; Mitrou et al. 2015a; Mitrou et al. 2015b; Mitrou et al. 2010; Mohammadpourhodki and Sargolzaei 2019; Nazni et al. 2015; Östman et al. 2005; Panetta et al. 2013; Park et al. 2014; Bashiri et al. 2014; Salbe et al. 2009; Thinathayalan et al. 2019; Wang et al. 2007; White and Johnston 2007; Yoon et al. 2012; Zeshan Ali et al. 2019) with 1781 individuals, have examined the health effects of vinegar consumption compared to a control group. The clinical outcomes evaluated encompassed FBG, PPG, HbA1C, FPI, PPI, HOMA‐IR, HDL, LDL, TC, TG, weight, BMI, WC, SBP, and DBP. There were 22 included clinical trials (Abid et al. 2020; Ali et al. 2018; Ebrahimi‐Mamaghani et al. 2009; Gheflati et al. 2019; Golzarand et al. 2008; Golzarand et al. 2009; Halima et al. 2017; Hamer and Van Loon 2012; Jafarirad et al. 2023; Johnston et al. 2004; Johnston et al. 2010; Johnston et al. 2009; Kahraman et al. 2011; Kausar et al. 2019; Liatis et al. 2010; Mahmoodi et al. 2013; Mitrou et al. 2015a, 2015b; Mohammadpourhodki and Sargolzaei 2019; Nazni et al. 2015; Bashiri et al. 2014; White and Johnston 2007; Yoon et al. 2012) involving patients diagnosed with type two diabetes mellitus (T2DM). Four trials (Jasbi et al. 2019; Khezri et al. 2018; Kondo, Kishi, Fushimi, and Kaga 2009; Park et al. 2014) focused on individuals classified as overweight or obese, eight trials (Chiu et al. 2020; Derakhshandeh‐Rishehri et al. 2014; Johnston and Buller 2005; Johnston et al. 2013; Östman et al. 2005; Panetta et al. 2013; Salbe et al. 2009; Thinathayalan et al. 2019) were conducted with healthy populations, and four studies (Mitrou et al. 2015a, 2015b; Wang et al. 2007; Zeshan Ali et al. 2019) addressed various other medical conditions. The primary studies were published between the years 2004 and 2023. Among the 38 RCTs reviewed, 24 investigated the effects of apple cider vinegar (ACV) (Abid et al. 2020; Chiu et al. 2020; Ebrahimi‐Mamaghani et al. 2009; Gheflati et al. 2019; Golzarand et al. 2008; Golzarand et al. 2009; Halima et al. 2017; Jafarirad et al. 2023; Johnston and Buller 2005; Johnston et al. 2004; Johnston et al. 2013; Johnston et al. 2010; Johnston et al. 2009; Kausar et al. 2019; Khezri et al. 2018; Kondo, Kishi, Fushimi, and Kaga 2009; Mahmoodi et al. 2013; Mohammadpourhodki and Sargolzaei 2019; Nazni et al. 2015; Panetta et al. 2013; Bashiri et al. 2014; Salbe et al. 2009; Thinathayalan et al. 2019; White and Johnston 2007), while three studies concentrated on wine vinegar (Jasbi et al. 2019; Liatis et al. 2010; Mitrou et al. 2015a, 2015b), two on white vinegar (Hamer and Van Loon 2012; Östman et al. 2005), one on ginseng vinegar (Yoon et al. 2012), one on grape vinegar (Kahraman et al. 2011), two on date vinegar (Ali et al. 2018; Zeshan Ali et al. 2019), one on honey vinegar (Derakhshandeh‐Rishehri et al. 2014), one on pomegranate vinegar (Park et al. 2014), and one on cranberry vinegar (Wang et al. 2007). Furthermore, two studies did not specify the type of vinegar utilized. The duration of intervention ranged from 1 day to 12 weeks. Besides, the administration of vinegar varied between 1.5 and 60 mL/d. In the control group, seven trials (Gheflati et al. 2019; Golzarand et al. 2008, 2009; Park et al. 2014; Wang et al. 2007; Yoon et al. 2012; Zeshan Ali et al. 2019) used a placebo, nine (Derakhshandeh‐Rishehri et al. 2014; Halima et al. 2017; Mahmoodi et al. 2013; Mitrou et al. 2015a, 2015b; Panetta et al. 2013; Salbe et al. 2009; Thinathayalan et al. 2019) employed water, 16 studies (Abid et al. 2020; Ali et al. 2018; Chiu et al. 2020; Hamer and Van Loon 2012; Jafarirad et al. 2023; Jasbi et al. 2019; Johnston and Buller 2005; Johnston et al. 2004; Johnston et al. 2013; Johnston et al. 2010; Kausar et al. 2019; Khezri et al. 2018; Kondo, Kishi, Fushimi, and Kaga 2009; Liatis et al. 2010; Östman et al. 2005; White and Johnston 2007) utilized alternative control measures, and one trial had no treatment at all (Bashiri et al. 2014). Furthermore, five studies (Ebrahimi‐Mamaghani et al. 2009; Johnston et al. 2009; Kahraman et al. 2011; Mohammadpourhodki and Sargolzaei 2019; Nazni et al. 2015) did not mention their control groups.

### 3.3 Methodological Quality

The quality of the included meta‐analyses was evaluated using the AMSTAR 2 criteria. From the 10 included SRMAs, three were rated high (Arjmandfard et al. 2025; Shahinfar et al. 2022; Valdes et al. 2021), five were rated low (Cheng et al. 2020; Hadi et al. 2021; Shishehbor et al. 2017; Sohouli et al. 2022; Tehrani et al. 2025), and two (Fakhri et al. 2022; Siddiqui et al. 2018) were rated critically low in quality when assessed using the AMSTAR 2 tool (Table S6). The quality of the included RCTs was assessed using the Cochrane RoB tool. Out of the studies examined, three were of high quality (Jasbi et al. 2019; Park et al. 2014; Yoon et al. 2012), three were rated as fair (Kondo, Kishi, Fushimi, and Kaga 2009; Panetta et al. 2013; Zeshan Ali et al. 2019), and the remaining studies (Abid et al. 2020; Ali et al. 2018; Chiu et al. 2020; Derakhshandeh‐Rishehri et al. 2014; Ebrahimi‐Mamaghani et al. 2009; Gheflati et al. 2019; Golzarand et al. 2008, 2009; Halima et al. 2017; Hamer and Van Loon 2012; Jafarirad et al. 2023; Johnston and Buller 2005; Johnston et al. 2004, 2013, 2010, 2009; Kahraman et al. 2011; Kausar et al. 2019; Khezri et al. 2018; Liatis et al. 2010; Mahmoodi et al. 2013; Mitrou et al. 2015a, 2015b, 2010; Mohammadpourhodki and Sargolzaei 2019; Nazni et al. 2015; Östman et al. 2005; Bashiri et al. 2014; Salbe et al. 2009; Thinathayalan et al. 2019; Wang et al. 2007; White and Johnston 2007) were of low quality (Table S3).

### 3.4 Findings From the Umbrella Review

#### 3.4.1 Effect of Vinegar Consumption on FBG

**FIGURE 2:**
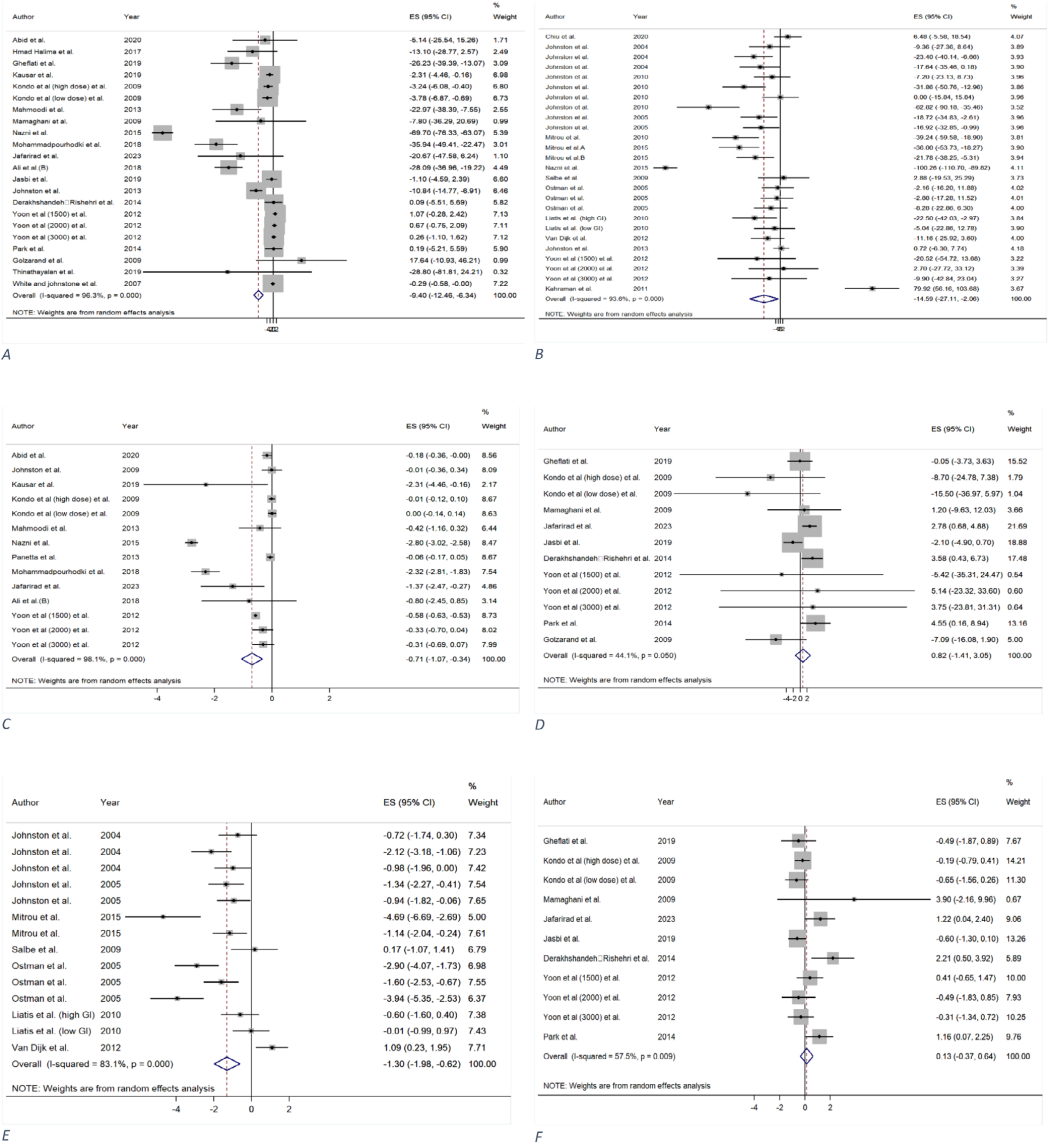
The effect of vinegar consumption on: (A) fasting blood glucose (FBG), (B) post prandial glucose (PPG), (C) hemoglobin A1c (HbA1c), (D) fasting plasma insulin (FPI), (E) post prandial insulin (PPI), (F) homeostatic model assessment of insulin resistance (HOMA‐IR).

A total of 19 RCTs (Abid et al. 2020; Ali et al. 2018; Derakhshandeh‐Rishehri et al. 2014; Ebrahimi‐Mamaghani et al. 2009; Gheflati et al. 2019; Golzarand et al. 2009; Halima et al. 2017; Jafarirad et al. 2023; Jasbi et al. 2019; Johnston et al. 2013; Kausar et al. 2019; Kondo, Kishi, Fushimi, and Kaga 2009; Mahmoodi et al. 2013; Mohammadpourhodki and Sargolzaei 2019; Nazni et al. 2015; Park et al. 2014; Thinathayalan et al. 2019; White and Johnston 2007; Yoon et al. 2012) from seven meta‐analyses (Arjmandfard et al. 2025; Cheng et al. 2020; Fakhri et al. 2022; Siddiqui et al. 2018; Sohouli et al. 2022; Tehrani et al. 2025; Valdes et al. 2021) showed that vinegar intake was associated with a significant reduction in FBG levels in comparison to the control group (MD = −9.40 mg/dL; 95% CI: −12.46, −6.34; *p* = < 0.001; GRADE = moderate) (Table 2 and Figure 2A). Substantial heterogeneity was observed across studies (*I* ^2^ = 96.3%; *p* = < 0.001). The heterogeneity between studies was explained by the type of vinegar and the subjects' health condition regarding FBG (Table S7). Subgroup analyses revealed a significant reduction in FBG levels among individuals with T2DM and overweight or obese patients. Among the various types of vinegar, ACV was effective in significantly lowering FBG levels. Additionally, subgroup analysis indicated that a duration of treatment > 8 weeks resulted in a significant reduction in FBG. Furthermore, both doses of vinegar (> 20 mL/d and < = 20 mL/d) significantly decreased FBG levels (Table S7). The visual inspection of funnel plot (Figure S1) and the results of Begg's test indicated that there was no statistically significant evidence of publication bias (*p* = 0.225).

**TABLE 2:** Summary of pooled effect estimates of vinegar consumption on glycemic, lipid, anthropometric, and blood pressure outcomes.

Outcome	No. of effect sizes	No. of participants (intervention/control)	Duration of intervention (Range)	Dose range (ml/d)	MD (95% CI)	*p*	Direction of effect	*I* ^2^ (%)	*p*‐heterogeneity	*p* for publication bias	Certainty of evidence^a^ (GRADE)
FBG	22	1345 (694/651)	2 days–12 weeks	1.5–60	−9.40 (−12.46, −6.34)	< 0.001	↓ (favorable)	96.3	< 0.001	0.225	Moderate
PPG	26	578 (289/289)	1 days–12 weeks	1.5–50	−14.59 (−27.11, −2.06)	0.022	↓ (favorable)	93.6	< 0.001	0.067	Moderate
HbA1c	14	969 (489/480)	4–12 weeks	1.5–60	−0.70 (−1.07, −0.33)	< 0.001	↓ (favorable)	98.1	< 0.001	0.037	Moderate
FPI	12	687 (335/352)	4–12 weeks	1.5–60	0.82 (−1.40, 3.05)	0.47	→ (no effect)	44.1	0.05	0.249	Moderate
PPI	14	278 (139/139)	1 days–2 weeks	18–40	−1.29 (−1.97, −0.61)	< 0.001	↓ (favorable)	83.1	< 0.001	0.05	High
HOMA‐IR	11	669 (336/333)	4–12 weeks	1.5–60	0.13 (−0.37, 0.64)	0.608	→ (no effect)	57.5	0.009	0.072	Moderate
HDL	17	1147 (597/550)	4–12 weeks	1.5–30	0.87 (−0.47, 2.22)	0.202	→ (no effect)	70	< 0.001	0.323	Moderate
LDL	18	1187 (617/570)	4–12 weeks	1.5–30	−8.18 (−18.46, 2.10)	0.119	→ (no effect)	96.1	< 0.001	0.272	Low
TG	18	1187 (617/570)	4–12 weeks	1.5–30	−14.94 (−30.59, 0.7)	0.061	→ (no effect)	95.7	< 0.001	0.063	High
TC	18	1187 (617/570)	4–12 weeks	1.5–30	−9.39 (−18.04, −0.75)	0.033	↓ (favorable)	90.4	< 0.001	0.025	Very low
Weight	13	918 (466/452)	5 days–12 weeks	1.5–60	−1.06 (−1.6, −0.52)	< 0.001	↓ (favorable)	11.1	0.334	0.903	High
BMI	15	900 (455/445)	4–12 weeks	1.5–60	−0.15 (−0.35, 0.04)	0.116	→ (no effect)	30.5	0.126	0.960	Moderate
WC	8	475 (239/236)	8–12 weeks	1.5–60	−0.91 (−1.92, 0.11)	0.080	→ (no effect)	61.8	0.010	0.999	Moderate
SBP	10	578 (295/283)	5 days–12 weeks	1.5–30	−2.94 (−4.81, −1.07)	0.002	↓ (favorable)	0	0.744	0.026	High
DBP	10	578 (295/283)	5 days–12 weeks	1.5–30	−0.00 (−2.09, 2.08)	0.997	→ (no effect)	64.9	0.002	0.956	Moderate

Abbreviations: BMI, body mass index; CI, confidence interval; DBP, diastolic blood pressure; FBG, fasting blood glucose; FPI, fasting plasma insulin; GRADE, Grading of Recommendations Assessment, Development, and Evaluation; HbA1c, hemoglobin A1c; HDL, high‐density lipoprotein; HOMA‐IR, homeostatic model assessment of insulin resistance; LDL, low‐density lipoprotein; PPG, post prandial glucose; PPI, post prandial insulin; SBP, systolic blood pressure; TC, Total cholesterol; TG, Triglycerides; wk., week.

^a^ Certainty of evidence was assessed using the GRADE approach.

#### 3.4.2 Effect of Vinegar Consumption on PPG

A total of 15 RCTs (Chiu et al. 2020; Hamer and Van Loon 2012; Johnston and Buller 2005; Johnston et al. 2004, 2013, 2010; Kahraman et al. 2011; Liatis et al. 2010; Mitrou et al. 2015a, 2015b, 2010; Nazni et al. 2015; Östman et al. 2005; Salbe et al. 2009; Yoon et al. 2012) included in four meta‐analyses (Shishehbor et al. 2017; Siddiqui et al. 2018; Sohouli et al. 2022; Tehrani et al. 2025) demonstrated that vinegar consumption significantly improved PPG levels in comparison to the control group (MD = −14.59 mg/dL; 95% CI: −27.11, −2.06; *p* = 0.022; GRADE = moderate) (Table 2 and Figure 2B). Considerable heterogeneity was identified among the studies (*I* ^2^ = 93.6%; *p* = < 0.001). Our subgroup analysis revealed that the heterogeneity observed between studies can be attributed to the type of vinegar and the health conditions (Table S7). The results from the subgroup analysis based on health condition, type of vinegar, and vinegar dosage showed that vinegar consumption significantly benefits healthy persons and other conditions. Among vinegar types, ACV and wine vinegar significantly reduced PPG. Besides, doses of < = 20 mL/d had a considerable effect on PPG (Table S7). The visual inspection of the funnel plot (Figure S2) and Begg's test did not indicate a significant publication bias (*p* = 0.067).

#### 3.4.3 Effect of Vinegar Consumption on HbA1C

Totally, 11 primary trials (Abid et al. 2020; Ali et al. 2018; Jafarirad et al. 2023; Johnston et al. 2009; Kausar et al. 2019; Kondo, Kishi, Fushimi, and Kaga 2009; Mahmoodi et al. 2013; Mohammadpourhodki and Sargolzaei 2019; Nazni et al. 2015; Panetta et al. 2013; Yoon et al. 2012) included from four meta‐analyses (Arjmandfard et al. 2025; Siddiqui et al. 2018; Sohouli et al. 2022; Tehrani et al. 2025) revealed that consuming vinegar had a significant effect on HbA1c levels, compared to a control group (MD = −0.70%; 95% CI: −1.07, −0.33; *p* = < 0.001; GRADE = moderate) (Table 2 and Figure 2C). Significant heterogeneity was among the primary trials (*I* ^*2*^ = 98.1%; *p* < 0.001). Based on subgroup analyses, the type of vinegar consumed may account for the heterogeneity detected between the studies (Table S7). In the subgroup analyses, a notable decrease in HbA1c levels was identified in patients with T2DM who underwent vinegar intervention. Specifically, ACV and ginseng vinegar demonstrated significant improvement in HbA1c levels. Furthermore, a dosage of 20 mL/d or less and administration over 8 weeks or less, was associated with a significant reduction in HbA1c (Table S7). Additionally, the visual inspection of the funnel plot (Figure S3) and Begg's test indicated the presence of publication bias (*p* = 0.037).

#### 3.4.4 Effect of Vinegar Consumption on FPI

A synthesis of nine studies (Derakhshandeh‐Rishehri et al. 2014; Ebrahimi‐Mamaghani et al. 2009; Gheflati et al. 2019; Golzarand et al. 2009; Jafarirad et al. 2023; Jasbi et al. 2019; Kondo, Kishi, Fushimi, and Kaga 2009; Park et al. 2014; Yoon et al. 2012) derived from five meta‐analyses (Arjmandfard et al. 2025; Fakhri et al. 2022; Siddiqui et al. 2018; Sohouli et al. 2022; Tehrani et al. 2025) revealed that the consumption of vinegar did not result in a statistically significant increase in FPI levels when compared to the control group (MD = 0.82 μu/mL; 95% CI: −1.40, 3.05; *p* = 0.470; GRADE = moderate) (Table 2 and Figure 2D). The studies presented a degree of homogeneity (*I* ^*2*^ = 44.1%; *p* = 0.050). Following the subgroup analysis, no significant improvement was seen for FPI in any of the subgroups (Table S7). Besides, the visual inspection of the funnel plot (Figure S4) and Egger's test showed no evidence of publication bias (*p* = 0.249).

#### 3.4.5 Effect of Vinegar Consumption on PPI

A total of eight studies (Hamer and Van Loon 2012; Johnston and Buller 2005; Johnston et al. 2004; Liatis et al. 2010; Mitrou et al. 2015a, 2015b; Östman et al. 2005; Salbe et al. 2009) within one meta‐analysis (Shishehbor et al. 2017) demonstrated that vinegar consumption significantly reduced PPI compared to the control group (MD = −1.29 mu/L; 95% CI: −1.97, −0.61; *p* = < 0.001; GRADE = high) (Table 2 and Figure 2E). Nonetheless, considerable heterogeneity was noted among the studies (*I* ^*2*^ = 83.1%; *p* = < 0.001). Subgroup analysis based on health status, kind of vinegar, and vinegar dosage was done. The type of vinegar consumed may account for the heterogeneity observed between studies in the subgroup analyses (Table S7). It has revealed a significant reduction in PPI with the vinegar intervention compared to the control group in healthy individuals and other conditions. Specifically, ACV markedly decreased PPI. Furthermore, both vinegar dosages > 20 mL/d and < = 20 mL/d significantly lowered PPI levels (Table S7). The visual examination of the funnel plot (Figure S5) and Egger's test indicated no significant publication bias (*p* = 0.050).

#### 3.4.6 Effect of Vinegar Consumption on HOMA‐IR

Totally, eight RCTs (Derakhshandeh‐Rishehri et al. 2014; Ebrahimi‐Mamaghani et al. 2009; Gheflati et al. 2019; Jafarirad et al. 2023; Jasbi et al. 2019; Kondo, Kishi, Fushimi, and Kaga 2009; Park et al. 2014; Yoon et al. 2012) of three meta‐analyses (Arjmandfard et al. 2025; Siddiqui et al. 2018; Sohouli et al. 2022) investigated HOMA‐IR with vinegar intake and reported a non‐meaningful rise (MD = 0.13; 95% CI: −0.37, 0.64; *p* = 0.608; GRADE = moderate) (Table 2 and Figure 2F). There was substantial heterogeneity among studies (*I* ^*2*^ = 57.5%; *p* = 0.009). Subgroup analysis based on intervention duration, vinegar type, vinegar dosage, and participants' health condition explained the heterogeneity observed between studies (Table S7). The subgroup analysis findings found no significant enhancement in HOMA‐IR across any of the subgroups (Table S7). The visual inspection of the funnel plot (Figure S6) and the findings of Egger's test were not statistically significant (*p* = 0.072).

#### 3.4.7 Effect of Vinegar Consumption on HDL

**FIGURE 3:**
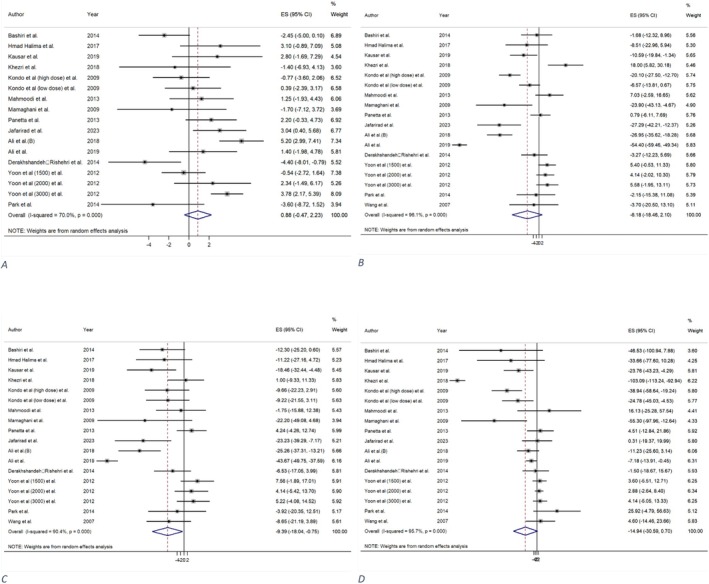
The effect of vinegar consumption on: (A) High‐density lipoprotein (HDL), (B) low‐density lipoprotein (LDL), (C) total cholesterol (TC), (D) triglycerides (TG).

The analysis of 14 studies (Ali et al. 2018; Derakhshandeh‐Rishehri et al. 2014; Ebrahimi‐Mamaghani et al. 2009; Halima et al. 2017; Jafarirad et al. 2023; Kausar et al. 2019; Khezri et al. 2018; Kondo, Kishi, Fushimi, and Kaga 2009; Mahmoodi et al. 2013; Panetta et al. 2013; Park et al. 2014; Bashiri et al. 2014; Yoon et al. 2012; Zeshan Ali et al. 2019) from four meta‐analyses (Arjmandfard et al. 2025; Siddiqui et al. 2018; Sohouli et al. 2022; Tehrani et al. 2025) suggested that vinegar consumption didn't result in a statistically significant elevation in HDL levels relative to the control group (MD = 0.87 mg/dL; 95% CI: −0.47, 2.22; *p* = 0.202; GRADE = moderate) (Table 2 and Figure 3A). The studies exhibited a level of heterogeneity (*I* ^2^ = 70%; *p* = < 0.001). The heterogeneity between trials can be explained by the type of vinegar and the health condition of participants (Table S7). The subgroup analysis indicated that this notable increase was exclusively observed in patients with T2DM. Besides, we did not find significant effects in other subgroups (Table S7). Furthermore, the visual inspection of the funnel plot (Figure S7) and Begg's test did not reveal statistically significant publication bias (*p* = 0.323).

#### 3.4.8 Effect of Vinegar Consumption on LDL

A total of 15 primary studies (Ali et al. 2018; Derakhshandeh‐Rishehri et al. 2014; Ebrahimi‐Mamaghani et al. 2009; Halima et al. 2017; Jafarirad et al. 2023; Kausar et al. 2019; Khezri et al. 2018; Kondo, Kishi, Fushimi, and Kaga 2009; Mahmoodi et al. 2013; Panetta et al. 2013; Park et al. 2014; Bashiri et al. 2014; Wang et al. 2007; Yoon et al. 2012; Zeshan Ali et al. 2019) from five meta‐analyses (Arjmandfard et al. 2025; Siddiqui et al. 2018; Sohouli et al. 2022; Tehrani et al. 2025; Valdes et al. 2021) concluded that vinegar consumption does not significantly reduce LDL levels compared to a control group (MD = −8.18 mg/dL; 95% CI: −18.46, 2.10; *p* = 0.119; GRADE = low) (Table 2 and Figure 3B). The investigations demonstrated significant heterogeneity (*I* ^*2*^ = 96.1%; *p* = < 0.001). The subgroup analyses show that the kind of vinegar taken and the individuals' health conditions could be responsible for the heterogeneity observed between studies (Table S7). Following subgroup analysis, a substantial reduction in LDL is observed in the ginseng vinegar intervention. We did not observe significant effects in other subgroups (Table S7). The visual examination of the funnel plot (Figure S8) and Egger's test indicated no significant publication bias (*p* = 0.272). Begg's test revealed no meaningful publication bias (*p* = 0.272).

#### 3.4.9 Effect of Vinegar Consumption on TC

The analysis of 15 trials (Ali et al. 2018; Derakhshandeh‐Rishehri et al. 2014; Ebrahimi‐Mamaghani et al. 2009; Halima et al. 2017; Jafarirad et al. 2023; Kausar et al. 2019; Khezri et al. 2018; Kondo, Kishi, Fushimi, and Kaga 2009; Mahmoodi et al. 2013; Panetta et al. 2013; Park et al. 2014; Bashiri et al. 2014; Wang et al. 2007; Yoon et al. 2012; Zeshan Ali et al. 2019) from five meta‐analyses (Arjmandfard et al. 2025; Siddiqui et al. 2018; Sohouli et al. 2022; Tehrani et al. 2025; Valdes et al. 2021) demonstrated that vinegar intake resulted in a substantial reduction in TC levels compared to the control group (MD = −9.39 mg/dL; 95% CI: −18.04, −0.75; *p* = 0.033, GRADE = very low) (Table 2 and Figure 3C). There was considerable heterogeneity between the studies (*I* ^*2*^ = 90.4%; *p* = < 0.001). In subgroup analysis, the heterogeneity between trials was elucidated by the type of vinegar and the health conditions (Table S7). The subgroup analysis revealed a significant reduction in TC levels among patients with T2DM who underwent the vinegar intervention, in contrast to the control group. Depending on the type of vinegar, ACV significantly lowered TC levels. Furthermore, we discovered that vinegar considerably decreased TC levels in durations exceeding 8 weeks. However, vinegar in both dose categories (> 20 mL/d and < = 20 mL/d) did not significantly affect TC (Table S7). The visual inspection of the funnel plot (Figure S9) and Begg's test revealed statistically significant evidence of publication bias (*p* = 0.025).

#### 3.4.10 Effect of Vinegar Consumption on TG

A total of 15 trials (Ali et al. 2018; Derakhshandeh‐Rishehri et al. 2014; Ebrahimi‐Mamaghani et al. 2009; Halima et al. 2017; Jafarirad et al. 2023; Kausar et al. 2019; Khezri et al. 2018; Kondo, Kishi, Fushimi, and Kaga 2009; Mahmoodi et al. 2013; Panetta et al. 2013; Park et al. 2014; Bashiri et al. 2014; Wang et al. 2007; Yoon et al. 2012; Zeshan Ali et al. 2019) from five meta‐analyses (Arjmandfard et al. 2025; Siddiqui et al. 2018; Sohouli et al. 2022; Tehrani et al. 2025; Valdes et al. 2021) indicated that vinegar consumption was linked to a non‐significant reduction in TG levels (MD = −14.94 mg/dL; 95% CI: −30.59, 0.7; *p* = 0.061; GRADE = high) (Table 2 and Figure 3D) compared to the control group. There was significant heterogeneity between the trials (*I* ^*2*^ = 95.7%; *p* = < 0.001). Subgroup analysis based on intervention duration, vinegar type, and subjects' health condition clarified the heterogeneity among studies (Table S7). In addition, no significant improvement in TG was observed in any subgroup (Table S7). The visual examination of the funnel plot (Figure S10) and Begg's test revealed no considerable publication bias (*p* = 0.063).

#### 3.4.11 Effect of Vinegar Consumption on Weight

**FIGURE 4:**
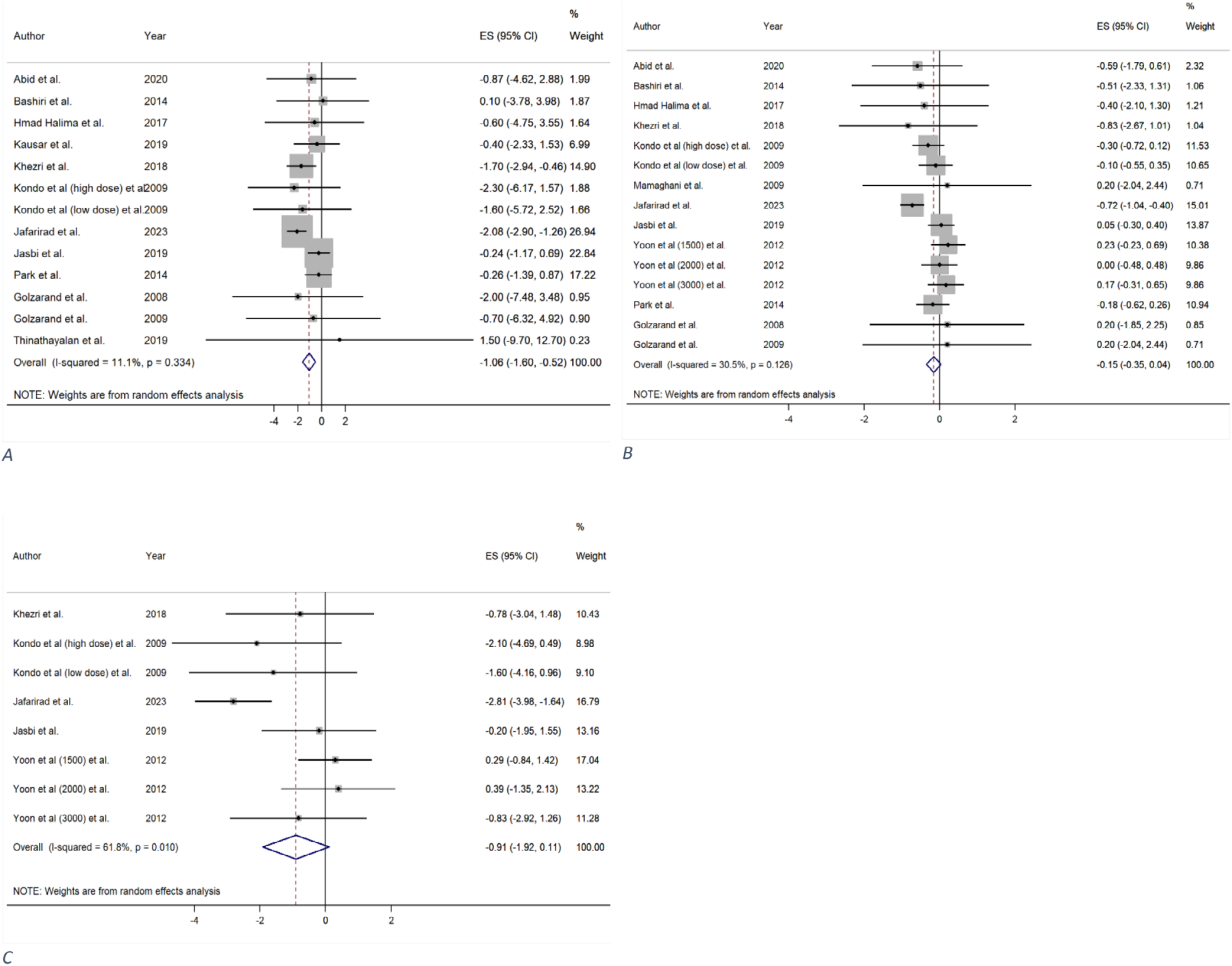
The effect of vinegar consumption on: (A) Weight, (B) body mass index (BMI), (C) waist circumference (WC).

A total of 12 trials (Abid et al. 2020; Golzarand et al. 2008; Golzarand et al. 2009; Halima et al. 2017; Jafarirad et al. 2023; Jasbi et al. 2019; Kausar et al. 2019; Khezri et al. 2018; Kondo, Kishi, Fushimi, and Kaga 2009; Park et al. 2014; Bashiri et al. 2014; Thinathayalan et al. 2019) from four meta‐analyses (Arjmandfard et al. 2025; Fakhri et al. 2022; Sohouli et al. 2022; Tehrani et al. 2025) examined the impact of vinegar intake on weight changes and reported a significant decrease (MD = −1.06 kg; 95% CI: −1.60, −0.52; *p* = < 0.001; GRADE = high) (Table 2 and Figure 4A). The studies exhibited low heterogeneity (*I* ^2^ = 11.1%; *p* = 0.334). Based on subgroup analysis, there is substantial weight reduction in T2DM and overweight or obese patients. Additionally, ACV showed a notable impact on weight in comparison to other types. Furthermore, doses > 20 mL/d and durations > 8 weeks resulted in significant weight changes (Table S7). The visual inspection of the funnel plot (Figure S11) and Begg's test indicated no statistically significant publication bias (*p* = 0.903).

#### 3.4.12 Effect of Vinegar Consumption on BMI

Analysis of 12 trials (Abid et al. 2020; Ebrahimi‐Mamaghani et al. 2009; Golzarand et al. 2008; Golzarand et al. 2009; Halima et al. 2017; Jafarirad et al. 2023; Jasbi et al. 2019; Khezri et al. 2018; Kondo, Kishi, Fushimi, and Kaga 2009; Park et al. 2014; Bashiri et al. 2014; Yoon et al. 2012) from five meta‐analyses (Arjmandfard et al. 2025; Fakhri et al. 2022; Siddiqui et al. 2018; Sohouli et al. 2022; Tehrani et al. 2025) revealed that vinegar consumption was associated with a non‐significant decrease in BMI relative to the control group (MD = −0.15 kg/m^2^; 95% CI: −0.35, 0.04; *p* = 0.116; GRADE = moderate) (Table 2 and Figure 4B). No significant heterogeneity was observed among the trials (*I* ^2^ = 30.5%; *p* = 0.126). The subgroup analysis indicated that the significant decrease observed was specific to the ACV type. We did not find any significant effects based on health condition categories (T2DM and overweight/obese), vinegar dosages (> 20 mL/d and < = 20 mL/d), or durations (> 8 weeks and < = 8 weeks) respectively (Table S7). Additionally, a visual inspection of the funnel plot (Figure S12) and Begg's test showed no signs of publication bias (*p* = 0.960).

#### 3.4.13 Effect of Vinegar Consumption on WC

Totally, five trials (Jafarirad et al. 2023; Jasbi et al. 2019; Khezri et al. 2018; Kondo, Kishi, Fushimi, and Kaga 2009; Yoon et al. 2012) across four meta‐analyses (Arjmandfard et al. 2025; Siddiqui et al. 2018; Sohouli et al. 2022; Tehrani et al. 2025) showed that vinegar drinking did not significantly reduce WC compared to the control (MD = −0.91 cm; 95% CI: −1.92, 0.11; *p* = 0.080; GRADE = moderate) (Table 2 and Figure 4C). Besides, considerable heterogeneity was observed among the investigations (*I* ^2^ = 61.8%; *p* = 0.010). The subgroup analyses indicated that vinegar type and dosage, individual health status, and intervention duration could account for the heterogeneity reported between studies (Table S7). Besides, subgroup analysis has demonstrated that ACV significantly reduced WC in different vinegars. Moreover, vinegar dosages exceeding 20 mL/d and durations exceeding 8 weeks considerably diminished WC size. However, vinegar in health condition categories (T2DM, Overweight/obese) did not significantly affect WC (Table S7). The visual examination of the funnel plot (Figure S13) and Egger's test revealed no substantial publication bias (*p* = 0.999).

#### 3.4.14 Effect of Vinegar Consumption on SBP and DBP

**FIGURE 5:**
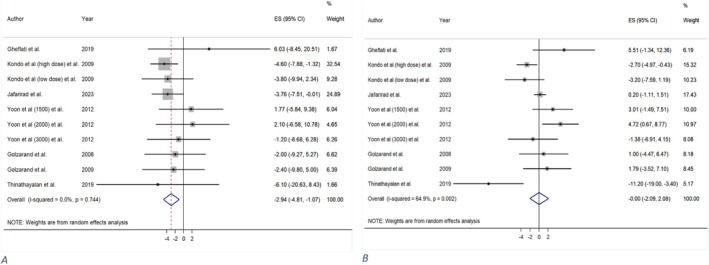
The effect of vinegar consumption on: (A) Systolic blood pressure (SBP), (B) diastolic blood pressure (DBP).

A total of seven primary trials (Gheflati et al. 2019; Golzarand et al. 2008; Golzarand et al. 2009; Jafarirad et al. 2023; Kondo, Kishi, Fushimi, and Kaga 2009; Thinathayalan et al. 2019; Yoon et al. 2012) from five meta‐analyses (Arjmandfard et al. 2025; Fakhri et al. 2022; Shahinfar et al. 2022; Siddiqui et al. 2018; Tehrani et al. 2025) supplied data regarding vinegar consumption and its effect on blood pressure. Vinegar significantly reduced SBP (MD = −2.94 mmHg; 95% CI: −4.81, −1.07; *p* = 0.002; GRADE = high) (Table 2 and Figure 5A), however, its effect on DBP was not notable (MD = −0.00 mmHg; CI: −2.09, 2.08; *p* = 0.997; GRADE = moderate) (Table 2 and Figure 5B). Furthermore, there was low (*I* ^2^ = 0%; *p* = 0.744) and high (*I* ^2^ = 64.9%; *p* = 0.002) heterogeneity among the trials for each outcome, separately. In the case of DBP, the observed heterogeneity was explained through subgroup analyses that considered variables such as the vinegar type, the participant's health status, and the intervention duration (Table S7). The subgroup analysis confirmed that vinegar significantly lowered SBP and DBP in overweight or obese people. Among the several varieties of vinegar, ACV significantly reduced SBP. However different types of vinegar did not considerably influence DBP. Moreover, an intake exceeding 20 mL/d of vinegar substantially decreased SBP. Although we did not see a significant effect of different doses of vinegar (> 20 and < = 20) on DBP. Furthermore, both SBP and DBP were considerably influenced by vinegar throughout a period exceeding 8 weeks of intervention (Table S7). The visual examination of the funnel plot (Figure S14) and Egger's test results for SBP were statistically significant (*p* = 0.026); however, the visual inspection of the funnel plot (Figure S15) and Egger's test findings indicated no substantial publication bias for DBP (*p* = 0.956).

## 4 Discussion

The current umbrella meta‐analysis focuses on the effect of vinegar on several health outcomes, including glycemic control, lipid profile, blood pressure, and anthropometric indicators. Pooled analysis suggests that several cardiometabolic factors such as FBG, PPG, PPI, HbA1c, TC, body weight, and SBP may improve following vinegar consumption, with more pronounced effects observed in subjects who consumed ACV. On the other hand, certain parameters, such as FPI, HOMA‐IR, LDL, HDL, TG, BMI, WC, and DBP, did not change significantly.

### 4.1 Glycemic Indices

Vinegar consumption showed consistent and clinically relevant benefits in blood glucose management, lowering FBG, PPG, and HbA1c. These effects were evident in individuals with T2DM and in interventions using ACV or wine vinegar at doses below 20 mL/day, and were supported by moderate to high certainty of evidence according to GRADE. In contrast, no significant changes were observed in FPI and HOMA‐IR despite moderate‐certainty evidence, suggesting that vinegar has a limited impact on insulin sensitivity indices. Previous studies have demonstrated that vinegar, particularly ACV, exerts heterogeneous effects on glycemic indices. Several meta‐analyses reported significant reductions in FBG and HbA1c, whereas FPI and HOMA‐IR showed no meaningful changes (Arjmandfard et al. 2025; Cheng et al. 2020; Hadi et al. 2021). However, another meta‐analysis failed to demonstrate improvements in FBS or PPG (Siddiqui et al. 2018).

It seems that acetic acid slows stomach emptying and delays carbs from entering the bloodstream and flattens glucose spikes (Johnston et al. 2004; Leeman et al. 2005). It also impairs enzymatic activities for hydrolyzing polysaccharides in the gastrointestinal tract (Noh et al. 2020). At the molecular level, acetic acid increases the adenosine monophosphate‐activated protein kinase (AMPK) pathway activity and helps glucose uptake by increasing glucose transporter type 4 (GLUT4) expression (Fushimi et al. 2006; Sakakibara et al. 2006). Additionally, acetic acid inhibits hepatic gluconeogenesis by suppressing related key enzymes like phosphoenolpyruvate carboxy‐kinase (PEPCK) and glucose‐6‐phosphatase (Kondo, Kishi, Fushimi, and Kaga 2009; Östman et al. 2005).

Vinegar consumption significantly reduced PPI, suggesting improved glucose metabolism and reduced insulin demand, potentially through enhanced insulin receptor substrate and phosphoinositide 3‐kinase/protein kinase B signaling (Mitrou et al. 2010). However, FPI and HOMA‐IR showed no significant changes, likely due to their slower responsiveness, modest baseline insulin resistance among participants, and mechanisms independent of insulin action, such as delayed gastric emptying and modulation of glucose transport.

### 4.2 Lipid Profile

Vinegar consumption significantly reduced TC, though this finding was supported by very low‐certainty evidence. Effects on TGs, HDL, and LDL were non‐significant and supported by low‐to‐high certainty ratings. However, the reduction in TC warrants cautious interpretation due to evidence of significant publication bias, as indicated by funnel plot asymmetry and Begg's test, alongside very low certainty of evidence and substantial heterogeneity. Previous meta‐analyses have shown that vinegar can significantly reduce TC and LDL concentrations, while demonstrating no statistically significant effects on other lipid parameters (Cheng et al. 2020; Hadi et al. 2021).

Several proposed mechanisms exist in this context. Acetic acid may reduce lipid synthesis by inhibiting fatty acid synthase, acetyl‐CoA carboxylase, and sterol regulatory element‐binding protein (A. Kondo et al. 2017; Wakil and Abu‐Elheiga 2009), while promoting fatty acid oxidation via promoting peroxisome proliferator‐activated receptor alpha and enhancing lipid breakdown through AMPK activation (Fushimi et al. 2006; Kondo, Kishi, Fushimi, and Kaga 2009).

### 4.3 Anthropometric Outcomes

Vinegar consumption was associated with a significant reduction in body weight, supported by high‐certainty evidence, particularly among overweight, obese, and diabetic populations consuming ACV at doses greater than 20 mL/day for durations exceeding 8 weeks. In contrast, no significant effects were observed for BMI or WC, for which the certainty of evidence was moderate. Conversely, the meta‐analysis conducted by Castagna et al. reported significant reductions in body weight, BMI, and WC following vinegar intake (Castagna et al. 2025), whereas another meta‐analysis found no statistically significant effect on WC (Sohouli et al. 2022).

Vinegar may help weight reduction through appetite suppression and increasing satiety by stimulating gut hormone secretion, namely glucagon‐like peptide‐1 (GLP‐1) and peptide YY (PYY), which reduce appetite (Cani et al. 2006). It may also cross the blood–brain barrier and influence the hypothalamus to regulate energy intake (Frost et al. 2014). Vinegar may also promote lipolysis and energy expenditure by increasing AMPK activity (Fushimi et al. 2006). Although vinegar consumption led to a significant reduction in body weight, no significant effect on BMI was observed, likely due to differences in the study populations assessed for these two outcomes.

### 4.4 Blood Pressure

This study demonstrated that vinegar consumption significantly reduced SBP, supported by high‐certainty evidence, but exerted no significant effect on DBP, which was underpinned by moderate certainty. These findings align with prior dose–response meta‐analyses reporting 3 mmHg SBP reductions at 30 mL/day vinegar doses (Shahinfar et al. 2022).

The antihypertensive effect of vinegar is mostly attributed to acetic acid, which can inhibit the renin‐angiotensin‐aldosterone system (RAAS), leading to reduced angiotensin II and aldosterone (S. Kondo et al. 2001). It also improves endothelial function and vasodilation via AMPK‐mediated upregulation of endothelial nitric oxide synthase (Petsiou et al. 2014). The lack of vinegar effect on DBP might be explained as the vinegar mechanisms of action mostly influence arterial stiffness and cardiac output rather than peripheral vascular resistance.

### 4.5 Strengths and Limitations

This umbrella review offers the most comprehensive evaluation of vinegar's health effects, encompassing both acute and chronic metabolic outcomes. Unlike prior studies, which either omitted GRADE assessments or reported only low‐to‐moderate certainty, we applied GRADE evaluations alongside subgroup analyses to better elucidate sources of heterogeneity.

Several limitations should be acknowledged. Three of ten meta‐analyses exhibited low methodological quality, as indicated by AMSTAR assessments and high RoB ratings, particularly due to inadequate blinding in over half of the RCTs, potentially introducing performance and detection biases. Moreover, small sample sizes reduced statistical power, dose–response analyses were lacking in some studies, and despite subgroup analyses revealing significant differences driven by variations in health status, vinegar type, dosage, and intervention duration across glycemic, lipid, and anthropometric outcomes (Abid et al. 2020; Ali et al. 2018; Derakhshandeh‐Rishehri et al. 2014; Gheflati et al. 2019; Kausar et al. 2019; Nazni et al. 2015; Park et al. 2014), high overall heterogeneity remained unresolved.

### 4.6 Clinical Implications and Future Research

Vinegar, especially ACV, offers a safe, cost‐effective adjunct for improving cardiometabolic parameters, particularly in overweight individuals or those with impaired glycemic control, at doses of 15–30 mL/day (typically diluted and consumed before meals). However, caution is advised due to potential adverse effects like gastrointestinal irritation, dental erosion, or reflux exacerbation, especially in patients with chronic kidney disease or those on glucose/potassium‐altering medications.

Future research should compare vinegar types, establish dose–response relationships in diverse metabolic populations, extend trials beyond 12 weeks, and investigate mechanisms such as AMPK activation, GLUT4 translocation, and gut microbiome effects.

## 5 Conclusion

The current umbrella meta‐analysis indicates that vinegar consumption was associated with a favorable impact on cardiometabolic risk factors, particularly key glycemic indices, cholesterol, weight, and SBP. However, no consistent beneficial effects were observed for other parameters. These effects might be mediated by cellular mechanisms such as delayed gastric emptying, enhanced insulin signaling, inhibition of lipid synthesis, and RAAS modulation. Although heterogeneity was observed in outcomes due to vinegar type, vinegar dose, intervention duration, and subjects' baseline metabolic status, and evidence for some outcomes remains inconclusive, it seems that vinegar can serve as a practical, low‐cost adjunct in metabolic health control.

## Author Contributions

**Reza Amiri Khosroshahi:** validation, methodology, software, conceptualization, formal analysis. **Hamed Mohammadi:** writing – review and editing, visualization, funding acquisition, project administration, resources. **Hoda Derakhshanian:** writing – original draft, writing – review and editing. **Forough Shahmohammadi:** writing – review and editing, investigation, data curation, writing – original draft, conceptualization, methodology, supervision.

## Funding

This work was supported by the Students' Scientific Research Center (SSRC) at Tehran University of Medical Sciences under Grant (grant number: 0‐0‐212‐91788).

## Ethics Statement

The authors have nothing to report.

## Conflicts of Interest

The authors declare no conflicts of interest.

## Supporting information


**Figure S1:** Funnel plot of FBG.
**Figure S2:** Funnel plot of PPG.
**Figure S3:** Funnel plot of HbA1C.
**Figure S4:** Funnel plot of FPI.
**Figure S5:** Funnel plot of PPI.
**Figure S6:** Funnel plot of HOMA‐IR.
**Figure S7:** Funnel plot of HDL.
**Figure S8:** Funnel plot of LDL.
**Figure S9:** Funnel plot of TC.
**Figure S10:** Funnel plot of TG.
**Figure S11:** Funnel plot of weight.
**Figure S12:** Funnel plot of BMI.
**Figure S13:** Funnel plot of WC.
**Figure S14:** Funnel plot of SBP.
**Figure S15:** Funnel plot of DBP.
**Table S1:** Preferred Reporting Items for Overviews of Reviews (PRIOR) checklist.
**Table S2:** Search strategy.
**Table S3:** Cochrane Risk of Bias Assessment.
**Table S4:** The Grading of Recommendations Assessment, Development and Evaluation (GRADE) quality of evidence for each outcome.
**Table S5:** Articles excluded during full text assessment and reasons for exclusion.
**Table S6:** Methodological quality of included systematic reviews and meta‐analyses using AMSTAR2.
**Table S7:** Subgroup analysis for vinegar consumption and health.

## Data Availability

The data that support the findings of this study are available from the corresponding author upon reasonable request.
