# Supplementary material for: Vinegar Consumption and Health: An Umbrella Review of Meta‐Analyses of Randomized Controlled Trials
Source: Food Sci Nutr. 2026 May 6;14(5):e71849. doi: 10.1002/fsn3.71849 (PMC13149749; doi:10.1002/fsn3.71849)
Supplement: Supplementary file 1 — Figure S1: Funnel plot of FBG. Figure S2: Funnel plot of PPG. Figure S3: Funnel plot of HbA1C. Figure S4: Funnel plot of FPI. Figure S5: Funnel plot of PPI. Figure S6: Funnel plot of HOMA‐IR. Figure S7: Funnel plot of HDL. Figure S8: Funnel plot of LDL. Figure S9: Funnel plot of TC. Figure S10: Funnel plot of TG. Figure S11: Funnel plot of weight. Figure S12: Funnel plot of BMI. Figure S13: Funnel plot of WC. Figure S14: Funnel plot of SBP. Figure S15: Funnel plot of DBP. Table S1: Preferred Reporting Items for Overviews of Reviews (PRIOR) checklist. Table S2: Search strategy. Table S3: Cochrane Risk of Bias Assessment. Table S4: The Grading of Recommendations Assessment, Development and Evaluation (GRADE) quality of evidence for each outcome. Table S5: Articles excluded during full text assessment and reasons for exclusion. Table S6: Methodological quality of included systematic reviews and meta‐analyses using AMSTAR2. Table S7: Subgroup analysis for vinegar consumption and health. [file FSN3-14-e71849-s001.docx]

**Vinegar consumption and health: An umbrella review of meta-analyses of randomized controlled trials (RCTs)**

Shahmohammadi et al.

Online Supplementary information

Supplementary data, including 7 supplementary Tables & 15 supplementary Figures

**Supplementary Figure legends:**

**Figure S1**. Funnel plot of FBG

**Figure S2**. Funnel plot of PPG

**Figure S3**. Funnel plot of HbA1C

**Figure S4**. Funnel plot of FPI

**Figure S5**. Funnel plot of PPI

**Figure S6**. Funnel plot of HOMA-IR

**Figure S7**. Funnel plot of HDL

**Figure S8**. Funnel plot of LDL

**Figure S9**. Funnel plot of TC

**Figure S10**. Funnel plot of TG

**Figure S11**. Funnel plot of weight

**Figure S12**. Funnel plot of BMI

**Figure S13**. Funnel plot of WC

**Figure S14**. Funnel plot of SBP

**Figure S15**. Funnel plot of DBP

**Table S1.** Preferred Reporting Items for Overviews of Reviews (PRIOR) checklist

| **Section**  Topic | **#** | **Item** | **Location reported** |
| --- | --- | --- | --- |
| **TITLE** | | | Page: |
| Title | 1 | Identify the report as an overview of reviews. | 1 |
| **ABSTRACT** | | |  |
| Abstract | 2 | Provide a comprehensive and accurate summary of the purpose, methods, and results of the overview of reviews. | 3 |
| **INTRODUCTION** | | |  |
| Rationale | 3 | Describe the rationale for conducting the overview of reviews in the context of existing knowledge. | 5,6 |
| Objectives | 4 | Provide an explicit statement of the objective(s) or question(s) addressed by the overview of reviews. | 5,6 |
| **METHODS** | | |  |
| Eligibility criteria | 5a | Specify the inclusion and exclusion criteria for the overview of reviews. If supplemental primary studies were included, this should be stated, with a rationale. | 7 |
|  | 5b | Specify the definition of ‘systematic review’ as used in the inclusion criteria for the overview of reviews. | 6 |
| Information sources | 6 | Specify all databases, registers, websites, organizations, reference lists, and other sources searched or consulted to identify systematic reviews and supplemental primary studies (if included).  Specify the date when each source was last searched or consulted. | 6 |
| Search strategy | 7 | Present the full search strategies for all databases, registers and websites, such that they could be reproduced. Describe any search filters and limits applied. | 6 |
| Selection process | 8a | Describe the methods used to decide whether a systematic review or supplemental primary study (if included) met the inclusion criteria of the overview of reviews. | 6,7 |
|  | 8b | Describe how overlap in the populations, interventions, comparators, and/or outcomes of systematic reviews was identified and managed during study selection. | 6,7 |
| Data collection process | 9a | Describe the methods used to collect data from reports. | 7 |
|  | 9b | If applicable, describe the methods used to identify and manage primary study overlap at the level  of the comparison and outcome during data collection. For each outcome, specify the method used to illustrate and/or quantify the degree of primary study overlap across systematic reviews. | 7 |
|  | 9c | If applicable, specify the methods used to manage discrepant data across systematic reviews during data collection. | 7 |
| Data items | 10 | List and define all variables and outcomes for which data were sought. Describe any assumptions made and/or measures taken to identify and clarify missing or unclear information. | 7 |
| Risk of bias assessment | 11a | Describe the methods used to *assess* risk of bias or methodological quality of the included systematic reviews. | 8 |
|  | 11b | Describe the methods used to *collect* data on (from the systematic reviews) and/or *assess* the risk of bias of the primary studies included in the systematic reviews. Provide a justification for instances where flawed, incomplete, or missing assessments are identified but not re-assessed. | 8 |
|  | 11c | Describe the methods used to *assess* the risk of bias of supplemental primary studies (if included). | Table S3 |
| Synthesis methods | 12a | Describe the methods used to summarize or synthesize results and provide a rationale for the choice(s). | 8 |
|  | 12b | Describe any methods used to explore possible causes of heterogeneity among results. | 8 |
|  | 12c | Describe any sensitivity analyses conducted to assess the robustness of the synthesized results. | 8 |
| Reporting bias assessment | 13 | Describe the methods used to *collect* data on (from the systematic reviews) and/or *assess* the risk of bias due to missing results in a summary or synthesis (arising from reporting biases at the levels of the systematic reviews, primary studies, and supplemental primary studies, if included). | 8 |
| Certainty assessment | 14 | Describe the methods used to *collect* data on (from the systematic reviews) and/or *assess* certainty (or confidence) in the body of evidence for an outcome. | 8,9 |
| **RESULTS** | | |  |
| Systematic review and supplemental primary study selection | 15a | Describe the results of the search and selection process, including the number of records screened, assessed for eligibility, and included in the overview of reviews, ideally with a flow diagram. | 9,10 |
|  | 15b | Provide a list of studies that might appear to meet the inclusion criteria, but were excluded, with the main reason for exclusion. | 9,10, Table S5 |
| **Section**  Topic | **#** | **Item** | **Location reported** |
| Characteristics of systematic reviews and supplemental primary studies | 16 | Cite each included systematic review and supplemental primary study (if included) and present its characteristics. | 9,10 |
| Primary study overlap | 17 | Describe the extent of primary study overlap across the included systematic reviews. | 7,9,10 |
| Risk of bias in systematic reviews, primary studies, and  supplemental primary studies | 18a | Present assessments of risk of bias or methodological quality for each included systematic review. | 10, Table S3, Table S6 |
|  | 18b | Present assessments (*collected* from systematic reviews or *assessed* anew) of the risk of bias of the primary studies included in the systematic reviews. | 10, Table S3 |
|  | 18c | Present assessments of the risk of bias of supplemental primary studies (if included). | 10, Table S3 |
| Summary or synthesis of results | 19a | For all outcomes, summarize the evidence from the systematic reviews and supplemental primary studies (if included). If meta-analyses were done, present for each the summary estimate and its precision and measures of statistical heterogeneity. If comparing groups, describe the direction of the effect. | 10-17 |
|  | 19b | If meta-analyses were done, present results of all investigations of possible causes of heterogeneity. | 10-17 |
|  | 19c | If meta-analyses were done, present results of all sensitivity analyses conducted to assess the robustness of synthesized results. | 10-17 |
| Reporting biases | 20 | Present assessments (*collected* from systematic reviews and/or *assessed* anew) of the risk of bias due to missing primary studies, analyses, or results in a summary or synthesis (arising from reporting biases at the levels of the systematic reviews, primary studies, and supplemental primary  studies, if included) for each summary or synthesis assessed. | 10, Table S6 |
| Certainty of evidence | 21 | Present assessments (*collected* or *assessed* anew) of certainty (or confidence) in the body of evidence for each outcome. | Table S4 |
| **DISCUSSION** | | |  |
| Discussion | 22a | Summarize the main findings, including any discrepancies in findings across the included systematic reviews and supplemental primary studies (if included). | 17,18 |
|  | 22b | Provide a general interpretation of the results in the context of other evidence. | 17-23 |
|  | 22c | Discuss any limitations of the evidence from systematic reviews, their primary studies, and supplemental primary studies (if included) included in the overview of reviews. Discuss any limitations of the overview of reviews methods used. | 17-23 |
|  | 22d | Discuss implications for practice, policy, and future research (both systematic reviews and primary research). Consider the relevance of the findings to the end users of the overview of reviews, e.g., healthcare providers, policymakers, patients, among others. | 17-23 |
| **OTHER INFORMATION** | | |  |
| Registration and protocol | 23a | Provide registration information for the overview of reviews, including register name and registration number, or state that the overview of reviews was not registered. | 6 |
|  | 23b | Indicate where the overview of reviews protocol can be accessed, or state that a protocol was not prepared. | 6 |
|  | 23c | Describe and explain any amendments to information provided at registration or in the protocol. Indicate the stage of the overview of reviews at which amendments were made. | 6 |
| Support | 24 | Describe sources of financial or non-financial support for the overview of reviews, and the role of the funders or sponsors in the overview of reviews. | 24 |
| Competing interests | 25 | Declare any competing interests of the overview of reviews' authors. | 24 |
| Author information | 26a | Provide contact information for the corresponding author. | 1 |
|  | 26b | Describe the contributions of individual authors and identify the guarantor of the overview of reviews. | 24 |
| Availability of data and other materials | 27 | Report which of the following are available, where they can be found, and under which conditions they may be accessed: template data collection forms; data collected from included systematic reviews and supplemental primary studies; analytic code; any other materials used in the overview of reviews. | 24 |

**Table S2.** Search strategy

| **Search strategy** | **Database**  **January 18 2025** |
| --- | --- |
| #1 ((vinegar[Title/Abstract] OR "Acetic Acid"[Title/Abstract] OR "glacial acetic acid"[Title/Abstract] OR "Acetic Acid"[Mesh] OR "apple cider vinegar"[Title/Abstract] OR "apple vinegar"[Title/Abstract] OR "apple acetic acid"[Title/Abstract] OR "apple acetate"[Title/Abstract] OR "apple glacial"[Title/Abstract] OR "apple ethanoic acid"[Title/Abstract] OR vinaigrette[Title/Abstract] OR "fermented foods"[Title/Abstract] OR Fermented[Title/Abstract] OR "AcOH"[Title/Abstract] OR "AcNA"[Title/Abstract] OR "dietary acetate"[Title/Abstract] OR "ACV"[Title/Abstract] OR "fermented apple"[Title/Abstract])  #2 ("Meta-Analysis"[Title/Abstract] OR "meta-analyses"[Title/Abstract] OR "Meta-Analysis"[Title/Abstract] OR "meta-analyze"[Title/Abstract] OR "Systematic Review"[Title/Abstract] OR "Systematic Review"[Publication Type] OR "Systematic Reviews as Topic"[MeSH] OR "Meta-Analysis as Topic"[MeSH] OR "Meta-Analysis"[Publication Type]))  #3 #1 AND #2 | **PubMed**  **451** |
| #1 (TITLE-ABS-KEY(vinegar) OR TITLE-ABS-KEY("Acetic Acid") OR TITLE-ABS-KEY("glacial acetic acid") OR TITLE-ABS-KEY("Acetic Acid") OR TITLE-ABS-KEY("apple cider vinegar") OR TITLE-ABS-KEY("apple vinegar") OR TITLE-ABS-KEY("apple acetic acid") OR TITLE-ABS-KEY("apple acetate") OR TITLE-ABS-KEY("apple glacial") OR TITLE-ABS-KEY("apple ethanoic acid") OR TITLE-ABS-KEY(vinaigrette) OR TITLE-ABS-KEY("fermented foods") OR TITLE-ABS-KEY(Fermented) OR TITLE-ABS-KEY("AcOH") OR TITLE-ABS-KEY("AcNA") OR TITLE-ABS-KEY("dietary acetate") OR TITLE-ABS-KEY("ACV") OR TITLE-ABS-KEY("fermented apple")  #2 TITLE-ABS-KEY("Meta-Analysis") OR TITLE-ABS-KEY("meta-analyses") OR TITLE-ABS-KEY("Meta-Analysis") OR TITLE-ABS-KEY("meta-analyze") OR TITLE-ABS-KEY("Systematic Review") OR TITLE-ABS-KEY("Systematic Review") OR TITLE-ABS-KEY("Systematic Reviews as Topic") OR TITLE-ABS-KEY("Meta-Analysis as Topic") OR TITLE-ABS-KEY("Meta-Analysis"))  #3 #1 AND #2 | **Scopus**  **1309** |
| #1 vinegar OR "Acetic Acid" OR "glacial acetic acid" OR "Acetic Acid" OR "apple cider vinegar" OR "apple vinegar" OR "apple acetic acid" OR "apple acetate" OR "apple glacial" OR "apple ethanoic acid" OR vinaigrette OR "fermented foods" OR Fermented OR "AcOH" OR "AcNA" OR "dietary acetate" OR "ACV" OR "fermented apple" (Topic)  #2 "Meta-Analysis" OR "meta-analyses" OR "Meta-Analysis" OR "meta-analyze" OR "Systematic Review" OR "Systematic Review" OR "Systematic Reviews as Topic" OR "Meta-Analysis as Topic" OR "Meta-Analysis" (Topic)  #3 #1 AND #2 | **ISI**  **704** |

**Table S3.** Cochrane Risk of Bias Assessment

| **Study** | **Random sequence generation** | **Allocation concealment** | **Blinding of participants**  **and personnel** | **Blinding of outcome**  **assessment** | **Incomplete**  **outcome data** | **Selective outcome**  **reporting** | **Other potential**  **threats to validity** | **General risk of bias** |
| --- | --- | --- | --- | --- | --- | --- | --- | --- |
| **Abid et al. 2020** | L | L | H | H | U | U | U | Poor |
| **Hmad Halima et al. 2017** | U | U | L | U | L | H | L | Poor |
| **Gheflati et al. 2019** | L | U | H | H | L | H | L | Poor |
| **Kausar et al. 2019** | L | U | H | H | L | H | L | poor |
| **Kondo et al. 2009** | L | U | L | L | L | L | U | Fair |
| **Mahmoodi et al. 2013** | H | U | L | U | L | H | L | poor |
| **Mamaghani et al. 2009** | L | U | U | U | L | U | U | Poor |
| **Nazni et al. 2015** | L | L | U | U | H | U | U | Poor |
| **Mohammadpourhodki et al. 2018** | U | U | H | H | L | H | L | Poor |
| **Jafarirad et al. 2023** | L | H | H | H | L | L | L | Poor |
| **Ali et al (B).2018** | L | U | L | U | L | U | L | Poor |
| **Jasbi et al. 2019** | L | L | U | L | L | L | L | Good |
| **Johnston et al. 2013** | U | U | L | U | H | U | U | Poor |
| **Derakhshandeh‑Rishehri et al. 2014** | U | U | U | U | L | L | L | Poor |
| **Yoon et al et al. 2012** | L | L | L | L | L | L | L | Good |
| **Park et al. 2014** | L | L | L | L | L | L | U | Good |
| **Golzarand et al. 2009** | L | U | H | U | U | U | U | Poor |
| **Golzarand et al. 2008** | L | U | H | U | L | L | U | Poor |
| **Thinathayalan et al. 2019** | L | U | U | L | U | U | U | Poor |
| **White and Johnstone et al. 2007** | L | U | H | U | U | L | L | Poor |
| **Chiu et al. 2020** | L | U | L | L | U | U | U | Poor |
| **Johnston et al. 2004** | L | U | H | H | H | U | U | Poor |
| **Johnston et al. 2005** | L | U | H | H | U | U | U | Poor |
| **Johnston et al. 2010** | L | U | L | L | U | U | U | Poor |
| **Mitrou et al. 2010** | L | U | H | U | U | L | H | Poor |
| **Mitrou et al (A). 2015** | L | U | H | U | U | U | U | Poor |
| **Mitrou et al (B). 2015** | L | U | H | U | U | U | U | Poor |
| **Salbe et al. 2009** | L | U | U | U | U | U | U | Poor |
| **Ostman et al. 2005** | L | U | H | U | U | L | U | Poor |
| **Liatis et al. 2010** | U | U | H | H | U | L | L | Poor |
| **Van Dijk et al. 2012** | L | U | H | H | U | L | H | Poor |
| **Kahraman et al. 2011** | U | U | H | H | U | L | H | Poor |
| **Johnston et al. 2009** | H | L | U | U | H | U | U | Poor |
| **Panetta et al. 2013** | L | L | L | L | L | U | U | Fair |
| **Bashiri et al. 2014** | L | U | U | U | L | U | L | Poor |
| **Khezri et al. 2018** | L | L | U | U | L | U | L | Poor |
| **Ali et al. 2019** | L | U | L | U | L | H | L | Fair |
| **Wang et al. 2007** | U | U | U | U | H | U | U | Poor |

L: low risk of bias; H: high risk of bias; U: unclear risk of bias

| **Table S4.** The Grading of Recommendations Assessment, Development and Evaluation (GRADE) quality of evidence for each outcome | | | | | | | | | | | | |
| --- | --- | --- | --- | --- | --- | --- | --- | --- | --- | --- | --- | --- |
| **Certainty assessment** | | | | | | | **№ of patients** | | **Effect** | | **Certainty** | **Importance** |
| **№ of studies** | **Study design** | **Risk of bias** | **Inconsistency** | **Indirectness** | **Imprecision** | **Other considerations** | **vinegar** | **control** | **Relative (95% CI)** | **Absolute (95% CI)** |  |  |
| **Fasting blood glucose (follow-up: mean 8 weeks)** | | | | | | | | | | | | |
| 19 | randomised trials | very serious^a^ | serious^b^ | not serious | not serious | all plausible residual confounding would reduce the demonstrated effect dose response gradient | 694 | 651 | - | MD **9.4 mg/dl lower** (12.46 lower to 6.34 lower) | ⨁⨁⨁◯ Moderate^a,b^ | CRITICAL |
| **Post prandial glucose (follow-up: mean 1 weeks)** | | | | | | | | | | | | |
| 15 | randomised trials | very serious^c^ | very serious^d^ | not serious | not serious | strong association all plausible residual confounding would reduce the demonstrated effect dose response gradient | 289 | 289 | - | MD **14.59 mg/dl lower** (27.11 lower to 2.06 lower) | ⨁⨁⨁◯  Moderate^c,d^ | CRITICAL |
| **Hemoglobin A1C (follow-up: mean 8 weeks)** | | | | | | | | | | | | |
| 11 | randomised trials | very serious^e^ | very serious^f^ | not serious | not serious | publication bias strongly suspected very strong association all plausible residual confounding would reduce the demonstrated effect dose response gradient^g^ | 489 | 480 | - | MD **0.71 % lower** (1.07 lower to 0.34 lower) | ⨁⨁⨁◯ Moderate^e,f,g^ | CRITICAL |
| **Fasting plasma insulin (follow-up: mean 8 weeks)** | | | | | | | | | | | | |
| 9 | randomised trials | very serious^h^ | not serious | not serious | serious^i^ | all plausible residual confounding would reduce the demonstrated effect dose response gradient | 355 | 352 | - | MD **0.82 μu/ml higher** (1.41 lower to 3.05 higher) | ⨁⨁⨁◯ Moderate^h,i^ | IMPORTANT |
| **Post prandial insulin (follow-up: mean 1 weeks)** | | | | | | | | | | | | |
| 8 | randomised trials | very serious^j^ | not serious^k^ | not serious | not serious | all plausible residual confounding would reduce the demonstrated effect dose response gradient | 139 | 139 | - | MD **1.3 mu/l lower** (1.98 lower to 0.62 lower) | ⨁⨁⨁⨁ High^j,k^ | IMPORTANT |
| **Homeostatic Model Assessment for Insulin Resistance (follow-up: mean 8 weeks)** | | | | | | | | | | | | |
| 8 | randomised trials | very serious^l^ | not serious^m^ | not serious | serious^n^ | all plausible residual confounding would reduce the demonstrated effect dose response gradient | 336 | 333 | - | MD **0.13 higher** (0.37 lower to 0.64 higher) | ⨁⨁⨁◯ Moderate^l,m,n^ | IMPORTANT |
| **High-density lipoprotein-cholesterol (follow-up: mean 8 weeks)** | | | | | | | | | | | | |
| 14 | randomised trials | very serious^o^ | not serious^p^ | not serious | serious^q^ | all plausible residual confounding would reduce the demonstrated effect dose response gradient | 597 | 550 | - | MD **0.88 mg/dl higher** (0.47 lower to 2.23 higher) | ⨁⨁⨁◯ Moderate^o,p,q^ | CRITICAL |
| **Low-density lipoprotein- cholesterol (follow-up: mean 8 weeks)** | | | | | | | | | | | | |
| 15 | randomised trials | very serious^r^ | very serious^s^ | not serious | serious^t^ | strong association all plausible residual confounding would reduce the demonstrated effect dose response gradient | 617 | 570 | - | MD **8.18 mg/dl lower** (18.46 lower to 2.1 higher) | ⨁⨁◯◯ Low^r,s,t^ | CRITICAL |
| **Triglycerides (follow-up: mean 8 weeks)** | | | | | | | | | | | | |
| 15 | randomised trials | very serious^u^ | not serious^v^ | not serious | serious^w^ | strong association all plausible residual confounding would reduce the demonstrated effect dose response gradient | 617 | 570 | - | MD **14.94 mg/dl lower** (30.59 lower to 0.7 higher) | ⨁⨁⨁⨁ High^u,v,w^ | CRITICAL |
| **Total cholesterol (follow-up: mean 8 weeks)** | | | | | | | | | | | | |
| 15 | randomised trials | very serious^x^ | very serious^y^ | not serious | not serious | publication bias strongly suspected strong association all plausible residual confounding would reduce the demonstrated effect^z^ | 617 | 570 | - | MD **9.39 mg/dl lower** (18.04 lower to 0.75 lower) | ⨁◯◯◯ Very low^x,y,z^ | CRITICAL |
| **Weight (follow-up: mean 8 weeks)** | | | | | | | | | | | | |
| 12 | randomised trials | very serious^aa^ | not serious | not serious | not serious | all plausible residual confounding would reduce the demonstrated effect dose response gradient | 466 | 452 | - | MD **1.06 kg lower** (1.6 lower to 0.52 lower) | ⨁⨁⨁⨁ High^aa^ | CRITICAL |
| **Body mass index (follow-up: mean 8 weeks)** | | | | | | | | | | | | |
| 12 | randomised trials | very serious^ab^ | not serious | not serious | serious^ac^ | all plausible residual confounding would reduce the demonstrated effect dose response gradient | 455 | 445 | - | MD **0.15 kg/m2 lower** (0.35 lower to 0.04 higher) | ⨁⨁⨁◯ Moderate^ab,ac^ | CRITICAL |
| **Waist circumference (follow-up: mean 8 weeks)** | | | | | | | | | | | | |
| 5 | randomised trials | very serious^ad^ | not serious^ae^ | not serious | serious^af^ | all plausible residual confounding would reduce the demonstrated effect dose response gradient | 239 | 236 | - | MD **0.91 cm lower** (1.92 lower to 0.11 higher) | ⨁⨁⨁◯ Moderate^ad,ae,af^ | IMPORTANT |
| **Systolic blood pressure (follow-up: mean 8 weeks)** | | | | | | | | | | | | |
| 7 | randomised trials | very serious^ag^ | not serious | not serious | not serious | publication bias strongly suspected strong association all plausible residual confounding would reduce the demonstrated effect dose response gradient^ah^ | 295 | 283 | - | MD **2.94 mmHg lower** (4.81 lower to 1.07 lower) | ⨁⨁⨁⨁ High^ag,ah^ | CRITICAL |
| **Diastolic blood pressure (follow-up: mean 8 weeks)** | | | | | | | | | | | | |
| 7 | randomised trials | very serious^ai^ | not serious^aj^ | not serious | serious^ak^ | all plausible residual confounding would reduce the demonstrated effect dose response gradient | 295 | 283 | - | MD **0 mmHg**  (2.09 lower to 2.08 higher) | ⨁⨁⨁◯ Moderate^ai,aj,ak^ | CRITICAL |
| **Abbreviations;** CI, confidence interval; MD, mean difference. | | | | | | | | | | | | |
| **Explanations**  a. More than 20% of RCTs for this outcome had a high risk of bias for at least one component of the Cochrane risk of bias tool. Those biases had a significant effect on the results of RCTs.  b. The I2 value was >50% (or Heterogeneity among the studies was high).  c. More than 20% of RCTs for this outcome had a high risk of bias for at least one component of the Cochrane risk of bias tool. Those biases had a significant effect on the results of RCTs.  d. Heterogeneity among the studies was high (I2>50%) and results were from moderate-to-low-quality evidence.  e. More than 20% of RCTs for this outcome had a high risk of bias for at least one component of the Cochrane risk of bias tool. Those biases had a significant effect on the results of RCTs.  f. Heterogeneity among the studies was high (I2>50%) and results were from moderate-to-low-quality evidence.  g. There is publication bias based on Begg's test (P = 0.037).  h. More than 20% of RCTs for this outcome had a high risk of bias for at least one component of the Cochrane risk of bias tool. Those biases had a significant effect on the results of RCTs.  i. The 95% CI derived from the pooled analysis for this outcome includes null value, indicating a lack of statistical significance (−1.41 to 3.05).  j. More than 20% of RCTs for this outcome had a high risk of bias for at least one component of the Cochrane risk of bias tool. Those biases had a significant effect on the results of RCTs.  k. The I2 value was >50%, however, the high heterogeneity was explained in the subgroup analyses.  l. More than 20% of RCTs for this outcome had a high risk of bias for at least one component of the Cochrane risk of bias tool. Those biases had a significant effect on the results of RCTs.  m. The I2 value was >50%, however, the high heterogeneity was explained in the subgroup analyses.  n. The 95% CI derived from the pooled analysis for this outcome includes null value, indicating a lack of statistical significance (−0.37 to 0.64).  o. More than 20% of RCTs for this outcome had a high risk of bias for at least one component of the Cochrane risk of bias tool. Those biases had a significant effect on the results of RCTs.  p. The I2 value was >50%, however, the high heterogeneity was explained in the subgroup analyses.  q. The 95% CI derived from the pooled analysis for this outcome includes null value, indicating a lack of statistical significance (−0.47 to 2.23).  r. More than 20% of RCTs for this outcome had a high risk of bias for at least one component of the Cochrane risk of bias tool. Those biases had a significant effect on the results of RCTs.  s. Heterogeneity among the studies was high (I2>50%) and results were from moderate-to-low-quality evidence.  t. The 95% CI derived from the pooled analysis for this outcome includes null value, indicating a lack of statistical significance (−8.18 to 2.10).  u. More than 20% of RCTs for this outcome had a high risk of bias for at least one component of the Cochrane risk of bias tool. Those biases had a significant effect on the results of RCTs.  v. The I2 value was >50%, however, the high heterogeneity was explained in the subgroup analyses.  w. The 95% CI derived from the pooled analysis for this outcome includes null value, indicating a lack of statistical significance (−30.59 to 0.70).  x. More than 20% of RCTs for this outcome had a high risk of bias for at least one component of the Cochrane risk of bias tool. Those biases had a significant effect on the results of RCTs.  y. Heterogeneity among the studies was high (I2>50%) and results were from moderate-to-low-quality evidence.  z. There is publication bias based on Begg's test (P = 0.025).  aa. More than 20% of RCTs for this outcome had a high risk of bias for at least one component of the Cochrane risk of bias tool. Those biases had a significant effect on the results of RCTs.  ab. More than 20% of RCTs for this outcome had a high risk of bias for at least one component of the Cochrane risk of bias tool. Those biases had a significant effect on the results of RCTs.  ac. The 95% CI derived from the pooled analysis for this outcome includes null value, indicating a lack of statistical significance (-0.35 to 0.04).  ad. More than 20% of RCTs for this outcome had a high risk of bias for at least one component of the Cochrane risk of bias tool. Those biases had a significant effect on the results of RCTs.  ae. The I2 value was >50%, however, the high heterogeneity was explained in the subgroup analyses.  af. The 95% CI derived from the pooled analysis for this outcome includes null value, indicating a lack of statistical significance (-1.92 to 0.11).  ag. More than 20% of RCTs for this outcome had a high risk of bias for at least one component of the Cochrane risk of bias tool. Those biases had a significant effect on the results of RCTs.  ah. There is publication bias based on Egger's test (P = 0.026).  ai. More than 20% of RCTs for this outcome had a high risk of bias for at least one component of the Cochrane risk of bias tool. Those biases had a significant effect on the results of RCTs.  aj. The I2 value was >50%, however, the high heterogeneity was explained in the subgroup analyses.  ak. The 95% CI derived from the pooled analysis for this outcome includes null value, indicating a lack of statistical significance (-2.09 to 2.08). | | | | | | | | | | | | |

| **Table S5.** Articles excluded during full text assessment and reasons for exclusion | | |
| --- | --- | --- |
| **Reason(s) for exclusion** | **Author, publication year (ref.)** |  |
| Not eligible intervention | Chan, 2023 (1) | **1** |
| Not eligible outcome | Hasan, 2022 (2) | **2** |
| Systematic review without meta-analysis | Launholt, 2020 (3) | **3** |
| Not eligible outcome | Wang, 2009 (4) | **4** |
| Not eligible outcome | Weber, 2023 (5) | **5** |
| Systematic review without meta-analysis | Astbury, 2024 (6) | **6** |
| Systematic review without meta-analysis | Brown, 2024 (7) | **7** |
| Not eligible outcome | DeSalvo, 2019 (8) | **8** |
| Not eligible intervention | Feng, 2024 (9) | **9** |
| Not eligible intervention | Gill, 2018 (10) | **10** |
| Systematic review without meta-analysis | Harrison, 2023 (11) | **11** |
| Not eligible intervention | Liu, 2024 (12) | **12** |
| Not eligible intervention | Marmitt, 2021 (13) | **13** |
| Not eligible intervention | Medina-Vera, 2021 (14) | **14** |
| Systematic review without meta-analysis | Neffe-Skocinska, 2023 (15) | **15** |
| Not eligible intervention | Sadia, 2018 (16) | **16** |
| Not eligible intervention | Shahrajabian, 2023 (17) | **17** |
| Not eligible intervention | Xavier, 2024 (18) | **18** |
| Systematic review without meta-analysis | Zhang, 2023 (19) | **19** |
| Ref, reference. | | |

| **Table S6.** Methodological quality of included systematic reviews and meta-analyses using AMSTAR2 | | | | | | | | | | | | | | | | | |
| --- | --- | --- | --- | --- | --- | --- | --- | --- | --- | --- | --- | --- | --- | --- | --- | --- | --- |
| **Author, year (ref.)** | **Q1** | **Q2** | **Q3** | **Q4** | **Q5** | **Q6** | **Q7** | **Q8** | **Q9** | **Q10** | **Q11** | **Q12** | **Q13** | **Q14** | **Q15** | **Q16** | **Level of evidence** |
| **Tehrani, 2023 (20)** | Yes | No | Yes | PY | Yes | Yes | Yes | Yes | Yes | No | Yes | No | Yes | Yes | Yes | Yes | Low |
| **Sohouli, 2022 (21)** | Yes | Yes | Yes | Yes | Yes | Yes | Yes | Yes | Yes | No | Yes | No | No | Yes | Yes | Yes | Low |
| **Shahinfar, 2022 (22)** | Yes | Yes | Yes | Yes | Yes | Yes | Yes | Yes | Yes | No | Yes | Yes | Yes | Yes | Yes | Yes | High |
| **Fakhri, 2022 (23)** | Yes | Yes | Yes | Yes | Yes | Yes | Yes | No | Yes | No | Yes | No | No | No | No | Yes | Critically low |
| **Cheng, 2020 (24)** | Yes | No | Yes | Yes | Yes | Yes | Yes | PY | Yes | Yes | Yes | Yes | Yes | Yes | Yes | Yes | Low |
| **Hadi, 2021 (25)** | Yes | No | Yes | Yes | Yes | Yes | Yes | Yes | Yes | No | Yes | No | Yes | Yes | Yes | Yes | Low |
| **Siddiqui, 2018 (26)** | Yes | No | Yes | Yes | Yes | Yes | Yes | Yes | Yes | Yes | Yes | No | No | Yes | No | Yes | Critically low |
| **Shishehbor, 2017 (27)** | No | No | No | Yes | Yes | Yes | Yes | Yes | PY | No | Yes | No | Yes | Yes | Yes | Yes | Low |
| **Arjmandfard, 2025 (28)** | Yes | Yes | Yes | Yes | Yes | Yes | Yes | Yes | Yes | Yes | Yes | Yes | Yes | Yes | Yes | Yes | High |
| **Valdes, 2021 (29)** | Yes | Yes | Yes | Yes | Yes | Yes | Yes | Yes | Yes | No | Yes | Yes | Yes | Yes | Yes | Yes | High |
| Ref, references. PY, partially yes. Q1: Did the research questions and inclusion criteria for the review include the components of PICO?, Q2: 2. Did the report of the review contain an explicit statement that the review methods were established prior to the conduct of the review and did the report justify any significant deviations from the protocol?; Q3, Did the review authors explain their selection of the study designs for inclusion in the review?; Q4, Did the review authors use a comprehensive literature search strategy?; Q5, Did the review authors perform study selection in duplicate?; Q6, Did the review authors perform data extraction in duplicate?; Q7, Did the review authors provide a list of excluded studies and justify the exclusions?; Q8, Did the review authors describe the included studies in adequate detail?; Q9, Did the review authors use a satisfactory technique for assessing the risk of bias?; Q10, Did the review authors report on the sources of funding?; Q11, Did the review authors use appropriate methods for statistical combination of results?; Q12, Did the review authors assess the potential impact of RoB in individual studies on the results?; Q13, Did the review authors account for RoB in individual studies when interpreting/ discussing the results of the review?; Q14, Did the review authors provide a satisfactory explanation for, and discussion of, any heterogeneity?; Q15, Did the review authors carry out an adequate investigation of publication bias?; Q16, Did the review authors report any potential sources of conflict of interest? | | | | | | | | | | | | | | | | | |

| **Table S7.** Subgroup analysis for vinegar consumption and health | | | | | | | |
| --- | --- | --- | --- | --- | --- | --- | --- |
| **Sub-grouped by** | **No. of effect size** | **Effect size^1^** | **95% CI** | **P-value** | **I^2^ (%)** | **P-heterogeneity^2^** | **P-between**  **subgroup heterogeneity^3^** |
| **FBG** | | | | | | | |
| Health condition | | | | | | | <0.001 |
| T2DM | 15 | -13.48 | -17.60, -9.35 | <0.001 | 97.3 | <0.001 |  |
| Overweight/obese | 4 | -2.55 | -4.25, -0.85 | 0.003 | 0.00 | 0.478 |  |
| Healthy | 3 | -6.39 | -16.64, 3.87 | 0.222 | 80.8 | 0.005 |  |
| Vinegar type | | | | | | | 0.003 |
| ACV | 15 | -15.75 | -22.43, -9.09 | <0.001 | 97.3 | <0.001 |  |
| Ginseng | 3 | 0.67 | -0.13, 1.46 | 0.099 | 0.00 | 0.709 |  |
| Other | 4 | -6.45 | -15.62, 2.71 | 0.167 | 91.3 | <0.001 |  |
| Vinegar dosage | | | | | | | 0.682 |
| > 20 | 11 | -12.02 | -21.52, -2.52 | 0.013 | 98 | <0.001 |  |
| <= 20 | 12 | -5.82 | -8.84, -2.81 | <0.001 | 89.5 | <0.001 |  |
| Duration | | | | | | | <0.001 |
| > 8 | 7 | -17.76 | -29.92, -5.60 | 0.004 | 98.5 | <0.001 |  |
| <= 8 | 15 | -1.26 | -2.98, 0.46 | 0.152 | 78.1 | <0.001 |  |
| **PPG** | | | | | | | |
| Health condition | | | | | | | <0.001 |
| T2DM | 11 | -17.51 | -48.22, 13.20 | 0.264 | 96.4 | <0.001 |  |
| Healthy | 11 | -7.34 | -13.62, -1.06 | 0.022 | 42.2 | 0.068 |  |
| Other | 4 | -23.38 | -45.45, -1.30 | 0.038 | 89.1 | <0.001 |  |
| Vinegar type | | | | | | | 0.002 |
| ACV | 13 | -21.26 | -41.44, -1.08 | 0.039 | 96.1 | <0.001 |  |
| Wine | 3 | -21.17 | -39.29, -3.05 | 0.022 | 65.8 | 0.054 |  |
| White | 4 | -5.99 | -13.21, 1.22 | 0.104 | 0 | 0.794 |  |
| Ginseng | 3 | -8.31 | -27.02, 10.40 | 0.384 | 0 | 0.606 |  |
| Other | 3 | 5.91 | -59.36, 71.17 | 0.859 | 96.9 | <0.001 |  |
| Vinegar dosage | | | | | | | <0.001 |
| > 20 | 9 | -15.67 | -48.15, 16.82 | 0.345 | 97.3 | <0.001 |  |
| <= 20 | 16 | -13.76 | -20.82, -6.69 | <0.001 | 61.7 | 0.001 |  |
| **HbA1c** | | | | | | | |
| Health condition | | | | | | | <0.001 |
| T2DM | 11 | -0.97 | -1.55, -0.39 | 0.001 | 97.9 | <0.001 |  |
| Other | 3 | -0.03 | -0.09, 0.04 | 0.441 | 98.1 | <0.001 |  |
| Vinegar type | | | | | | | <0.001 |
| ACV | 10 | -0.84 | -1.41, -0.27 | 0.004 | 98.6 | <0.001 |  |
| Ginseng | 3 | -0.48 | -0.67, -0.29 | <0.001 | 43.9 | 0.168 |  |
| Other | 1 | -0.80 | -2.45, 0.85 | 0.342 | - | - |  |
| Vinegar dosage | | | | | | | <0.001 |
| > 20 | 6 | -0.71 | -1.49, 0.07 | 0.073 | 99.1 | <0.001 |  |
| <= 20 | 8 | -0.68 | -1.08, -0.29 | 0.001 | 93.9 | <0.001 |  |
| Duration | | | | | | | <0.001 |
| > 8 | 7 | -0.75 | -1.56, 0.06 | 0.069 | 98.9 | <0.001 |  |
| <= 8 | 7 | -0.68 | -1.05, -0.32 | <0.001 | 95.3 | <0.001 |  |
| **FPI** | | | | | | | |
| Health condition | | | | | | | 0.100 |
| T2DM | 7 | 1.70 | -0.06, 3.46 | 0.058 | 0.00 | 0.440 |  |
| Overweight/obese | 4 | -1.16 | -7.18, 4.86 | 0.705 | 67.4 | 0.027 |  |
| Healthy | 1 | 3.58 | 0.43, 6.73 | 0.026 | - | - |  |
| Vinegar type | | | | | | | 0.975 |
| ACV | 6 | -0.51 | -4.20, 3.17 | 0.785 | 47.7 | 0.089 |  |
| Ginseng | 3 | 1.42 | -15.08, 17.93 | 0.866 | 0.00 | 0.863 |  |
| Other | 3 | 1.82 | -2.50, 6.14 | 0.408 | 79.5 | 0.008 |  |
| Vinegar dosage | | | | | | | 0.869 |
| > 20 | 5 | 0.14 | -3.30, 3.58 | 0.936 | 72.2 | 0.006 |  |
| <= 20 | 7 | 1.53 | -1.14, 4.20 | 0.262 | 0.00 | 0.513 |  |
| Duration | | | | | | | 0.056 |
| > 8 | 2 | -11.14 | -24.01, 1.73 | 0.090 | 0.00 | 0.619 |  |
| <= 8 | 10 | 1.21 | -0.91, 3.32 | 0.264 | 43 | 0.072 |  |
| **PPI** | | | | | | | |
| Health condition | | | | | | | <0.001 |
| T2DM | 5 | -0.32 | -1.16, 0.53 | 0.462 | 74.7 | 0.003 |  |
| Healthy | 7 | -1.56 | -2.42, -0.70 | <0.001 | 78.3 | <0.001 |  |
| Other | 2 | -3.26 | -5.76, -0.76 | 0.011 | 79.8 | 0.026 |  |
| Vinegar type | | | | | | | 0.806 |
| ACV | 6 | -1.03 | -1.56, -0.49 | <0.001 | 40.6 | 0.135 |  |
| Wine | 3 | -1.56 | -3.65, 0.53 | 0.143 | 88.4 | <0.001 |  |
| White | 4 | -1.79 | -3.97, 0.38 | 0.106 | 94.1 | <0.001 |  |
| Other | 1 | -1.14 | -2.04, -0.24 | 0.014 | - | - |  |
| Vinegar dosage | | | | | | | 0.523 |
| > 20 | 6 | -1.57 | -3.12, -0.02 | 0.048 | 91.3 | <0.001 |  |
| <= 20 | 8 | -1.17 | -1.75, -0.58 | <0.001 | 63.7 | 0.007 |  |
| **HOMA-IR** | | | | | | | |
| Health condition | | | | | | | 0.018 |
| T2DM | 6 | 0.15 | -0.51, 0.81 | 0.663 | 31.8 | 0.197 |  |
| Overweight/obese | 4 | -0.15 | -0.81, 0.51 | 0.655 | 63.2 | 0.043 |  |
| Healthy | 1 | 2.21 | 0.50, 3.92 | 0.011 | - | - |  |
| Vinegar type | | | | | | | 0.742 |
| ACV | 5 | -0.01 | -0.76, 0.73 | 0.977 | 51.7 | 0.082 |  |
| Ginseng | 3 | -0.08 | -0.73, 0.56 | 0.800 | 0.00 | 0.504 |  |
| Other | 3 | 0.79 | -0.85, 2.44 | 0.345 | 84.9 | 0.001 |  |
| Vinegar dosage | | | | | | | 0.970 |
| > 20 | 4 | 0.42 | -0.57, 1.41 | 0.402 | 78.1 | 0.003 |  |
| <= 20 | 7 | -0.01 | -0.61, 0.59 | 0.974 | 38.8 | 0.133 |  |
| Duration | | | | | | | 0.136 |
| > 8 | 2 | -0.33 | -0.83, 0.17 | 0.197 | 0.00 | 0.408 |  |
| <= 8 | 9 | 0.34 | -0.32, 1.01 | 0.312 | 61.2 | 0.008 |  |
| **HDL** | | | | | | | |
| Health condition | | | | | | | 0.017 |
| T2DM | 10 | 1.81 | 0.09, 3.53 | 0.039 | 72.4 | <0.001 |  |
| Overweight/obese | 4 | -0.70 | -2.46, 1.05 | 0.432 | 0.00 | 0.596 |  |
| Healthy | 2 | -0.97 | -7.43, 5.49 | 0.769 | 88.4 | 0.003 |  |
| Other | 1 | 1.40 | -1.98, 4.78 | 0.417 | - | - |  |
| Vinegar type | | | | | | | <0.001 |
| ACV | 10 | 0.74 | -0.61, 2.08 | 0.284 | 39.9 | 0.092 |  |
| Ginseng | 3 | 1.87 | -1.16, 4.91 | 0.226 | 79.5 | 0.008 |  |
| Other | 4 | -0.11 | -4.97, 4.75 | 0.964 | 87.9 | <0.001 |  |
| Vinegar dosage | | | | | | | 0.170 |
| > 20 | 6 | 0.29 | -1.89, 2.48 | 0.794 | 63.7 | 0.017 |  |
| <= 20 | 11 | 1.19 | -0.56, 2.94 | 0.182 | 73.4 | <0.001 |  |
| Duration | | | | | | | 0.289 |
| > 8 | 5 | 1.47 | -1.32, 4.26 | 0.300 | 72.6 | 0.006 |  |
| <= 8 | 12 | 0.62 | -0.98, 2.22 | 0.447 | 70.7 | <0.001 |  |
| **LDL** | | | | | | | |
| Health condition | | | | | | | <0.001 |
| T2DM | 10 | -6.55 | -14.67, 1.57 | 0.114 | 87 | <0.001 |  |
| Overweight/obese | 4 | -3.30 | -17.91, 11.31 | 0.658 | 89.5 | <0.001 |  |
| Healthy | 2 | -0.72 | -6.19, 4.75 | 0.796 | 0.00 | 0.482 |  |
| Other | 2 | -29.17 | -79.38, 19.96 | 0.241 | 96.9 | <0.001 |  |
| Vinegar type | | | | | | | <0.001 |
| ACV | 10 | -6.56 | -14.23, 1.12 | 0.094 | 82.9 | <0.001 |  |
| Ginseng | 3 | 4.99 | 1.27, 8.70 | 0.009 | 0.00 | 0.944 |  |
| Other | 5 | -18.52 | -43.16, 6.12 | 0.141 | 97.2 | <0.001 |  |
| Vinegar dosage | | | | | | | <0.001 |
| > 20 | 6 | -14.49 | -38.08, 9.09 | 0.228 | 98 | <0.001 |  |
| <= 20 | 11 | -4.41 | -11.03, 2.21 | 0.192 | 83.2 | <0.001 |  |
| NR | 1 | -3.70 | -20.50, 13.10 | 0.666 | - | - |  |
| Duration | | | | | | | 0.610 |
| > 8 | 6 | -8.96 | -20.20, 2.27 | 0.118 | 88.2 | <0.001 |  |
| <= 8 | 12 | -8.02 | -22.50, 6.46 | 0.278 | 97.2 | <0.001 |  |
| **TG** |  |  |  |  |  |  |  |
| Health condition | | | | | | | <0.001 |
| T2DM | 10 | -4.90 | -12.93, 3.13 | 0.232 | 60.1 | 0.007 |  |
| Overweight/obese | 4 | -36.32 | -91.23, 18.58 | 0.195 | 97.2 | <0.001 |  |
| Healthy | 2 | 1.47 | -10.73, 13.68 | 0.813 | 0.00 | 0.629 |  |
| Other | 2 | -4.80 | -14.07, 4.46 | 0.310 | 23.4 | 0.253 |  |
| Vinegar type | | | | | | | <0.001 |
| ACV | 10 | -30.57 | -62.41, 1.27 | 0.060 | 95.3 | <0.001 |  |
| Ginseng | 3 | 3.30 | -0.90, 7.50 | 0.124 | 0.00 | 0.971 |  |
| Other | 5 | -3.32 | -11.54, 4.91 | 0.429 | 34.9 | 0.188 |  |
| Vinegar dosage | | | | | | | <0.001 |
| > 20 | 6 | -24.52 | -64.36, 15.31 | 0.228 | 98.2 | <0.001 |  |
| <= 20 | 11 | -6.52 | -15.46, 2.41 | 0.153 | 67.8 | 0.001 |  |
| NR | 1 | 4.60 | -14.46, 23.66 | 0.636 | - | - |  |
| Duration | | | | | | | <0.001 |
| > 8 | 6 | -33.14 | -72.66, 6.38 | 0.100 | 97.2 | <0.001 |  |
| <= 8 | 12 | -0.27 | -6.27, 5.73 | 0.929 | 49.8 | 0.025 |  |
| **TC** | | | | | | | |
| Health condition | | | | | | | <0.001 |
| T2DM | 10 | -8.40 | -16.72, -0.08 | 0.048 | 76.3 | <0.001 |  |
| Overweight/obese | 4 | -4.89 | -11.09, 1.32 | 0.123 | 0.00 | 0.515 |  |
| Healthy | 2 | -0.68 | -11.20, 9.83 | 0.899 | 58.9 | 0.119 |  |
| Other | 2 | -26.61 | -60.91, 7.70 | 0.129 | 95.5 | <0.001 |  |
| Vinegar type | | | | | | | <0.001 |
| ACV | 10 | -8.36 | -14.38, -2.35 | 0.006 | 51.2 | 0.031 |  |
| Ginseng | 3 | 5.65 | 0.20, 11.09 | 0.042 | 0.00 | 0.878 |  |
| Other | 5 | -18.12 | -36.45, 0.22 | 0.053 | 93.2 | <0.001 |  |
| Vinegar dosage | | | | | | | <0.001 |
| > 20 | 6 | -13.00 | -32.07, 6.06 | 0.181 | 95.6 | <0.001 |  |
| <= 20 | 11 | -6.68 | -13.72, 0.37 | 0.063 | 70 | <0.001 |  |
| NR | 1 | -8.65 | -21.19, 3.89 | 0.176 | - | - |  |
| Duration | | | | | | | 0.754 |
| > 8 | 6 | -11.34 | -19.04, -3.64 | 0.004 | 58 | 0.036 |  |
| <= 8 | 12 | -8.33 | -20.68, 4.01 | 0.186 | 93.3 | <0.001 |  |
| **Weight** | | | | | | | |
| Health condition | | | | | | | 0.097 |
| T2DM | 7 | -1.68 | -2.38, -0.97 | <0.001 | 0 | 0.679 |  |
| Overweight/obese | 5 | -0.73 | -1.45, -0.01 | 0.046 | 17.6 | 0.303 |  |
| Healthy | 1 | 1.50 | -9.70, 12.70 | 0.793 | - | - |  |
| Vinegar type | | | | | | | 0.003 |
| ACV | 11 | -1.69 | -2.28, -1.09 | <0.001 | 0.00 | 0.928 |  |
| Other | 2 | -0.25 | -0.97, 0.47 | 0.498 | 0.00 | 0.979 |  |
| Vinegar dosage | | | | | | | 0.059 |
| > 20 | 8 | -1.34 | -2.11, -0.57 | 0.001 | 26.1 | 0.221 |  |
| <= 20 | 5 | -0.35 | -1.25, 0.55 | 0.445 | 0.00 | 0.979 |  |
| Duration | | | | | | | 0.527 |
| > 8 | 5 | -1.36 | -2.31, -0.42 | 0.005 | 0.00 | 0.819 |  |
| <= 8 | 8 | -0.86 | -1.75, 0.02 | 0.056 | 39.4 | 0.116 |  |
| **BMI** | | | | | | | |
| Health condition | | | | | | | 0.459 |
| T2DM | 10 | -0.15 | -0.50, 0.21 | 0.417 | 48.1 | 0.044 |  |
| Overweight/obese | 5 | -0.12 | -0.33, 0.08 | 0.238 | 0.00 | 0.688 |  |
| Vinegar type | | | | | | | 0.002 |
| ACV | 10 | -0.44 | -0.65, -0.23 | <0.001 | 0.00 | 0.658 |  |
| Ginseng | 3 | 0.14 | -0.14, 0.41 | 0.329 | 0.00 | 0.783 |  |
| Other | 2 | -0.04 | -0.31, 0.23 | 0.779 | 0.00 | 0.423 |  |
| Vinegar dosage | | | | | | | 0.013 |
| > 20 | 7 | -0.34 | -0.69, 0.01 | 0.056 | 46.1 | 0.084 |  |
| <= 20 | 8 | 0.01 | -0.20, 0.21 | 0.953 | 0.00 | 0.901 |  |
| Duration | | | | | | | 0.637 |
| > 8 | 5 | -0.24 | -0.53, 0.05 | 0.109 | 0.00 | 0.858 |  |
| <= 8 | 10 | -0.11 | -0.38, 0.17 | 0.447 | 51.6 | 0.029 |  |
| **WC** | | | | | | | |
| Health condition | | | | | | | 0.963 |
| T2DM | 4 | -0.77 | -2.49, 0.95 | 0.378 | 81.9 | 0.001 |  |
| Overweight/obese | 4 | -0.94 | -2.04, 0.16 | 0.094 | 0.00 | 0.630 |  |
| Vinegar type | | | | | | | 0.001 |
| ACV | 4 | -2.25 | -3.15, -1.35 | <0.001 | 0.00 | 0.429 |  |
| Ginseng | 3 | 0.12 | -0.74, 0.99 | 0.779 | 0.00 | 0.615 |  |
| Other | 1 | -0.20 | -1.95, 1.55 | 0.823 | - | - |  |
| Vinegar dosage | | | | | | | 0.003 |
| > 20 | 4 | -1.56 | -2.97, -0.16 | 0.029 | 56.5 | 0.075 |  |
| <= 20 | 4 | -0.05 | -0.87, 0.77 | 0.900 | 0.00 | 0.469 |  |
| Duration | | | | | | | 0.440 |
| > 8 | 3 | -1.43 | -2.84, -0.01 | 0.049 | 0.00 | 0.744 |  |
| <= 8 | 5 | -0.67 | -2.07, 0.73 | 0.346 | 76.7 | 0.002 |  |
| **SBP** | | | | | | | |
| Health condition | | | | | | | 0.357 |
| T2DM | 7 | -1.75 | -4.24, 0.73 | 0.167 | 0.00 | 0.698 |  |
| Overweight/obese | 2 | -4.42 | -7.32, -1.53 | 0.003 | 0.00 | 0.822 |  |
| Healthy | 1 | -6.10 | -20.63, 8.43 | 0.411 | - | - |  |
| Vinegar type | | | | | | | 0.079 |
| ACV | 7 | -3.70 | -5.75, -1.65 | <0.001 | 0.00 | 0.873 |  |
| Ginseng | 3 | 0.76 | -3.78, 5.31 | 0.742 | 0.00 | 0.809 |  |
| Vinegar dosage | | | | | | | 0.103 |
| > 20 | 5 | -3.91 | -6.11, -1.71 | 0.001 | 0.00 | 0.953 |  |
| <= 20 | 5 | -0.44 | -3.98, 3.10 | 0.808 | 0.00 | 0.625 |  |
| Duration | | | | | | | 0.189 |
| > 8 | 2 | -4.42 | -7.32, -1.53 | 0.003 | 0.00 | 0.822 |  |
| <= 8 | 8 | -1.88 | -4.33, 0.57 | 0.133 | 0.00 | 0.752 |  |
| **DBP** | | | | | | | |
| Health condition | | | | | | | <0.001 |
| T2DM | 7 | 1.51 | -0.19, 3.21 | 0.081 | 23.6 | 0.249 |  |
| Overweight/obese | 2 | -2.81 | -4.82, -0.79 | 0.006 | 0.00 | 0.843 |  |
| Healthy | 1 | -11.20 | -19.00, -3.40 | 0.005 | - | - |  |
| Vinegar type | | | | | | | 0.022 |
| ACV | 7 | -1.00 | -3.42, 1.42 | 0.418 | 65.4 | 0.008 |  |
| Ginseng | 3 | 2.54 | -0.78, 5.85 | 0.133 | 34.7 | 0.216 |  |
| Vinegar dosage | | | | | | | 0.079 |
| > 20 | 5 | -1.31 | -4.04, 1.43 | 0.349 | 69.0 | 0.012 |  |
| <= 20 | 5 | 1.60 | -1.82, 5.01 | 0.359 | 58.4 | 0.047 |  |
| Duration | | | | | | | 0.003 |
| > 8 | 2 | -2.81 | -4.82, -0.79 | 0.006 | 0.00 | 0.843 |  |
| <= 8 | 8 | 0.99 | -1.45, 3.43 | 0.428 | 58.6 | 0.018 |  |
| **Abbreviations**: CI, confidence interval  ^1^Calculated by Random-effects model  ^2^P heterogeneity within subgroup  ^3^P heterogeneity between subgroups | | | | | | | |


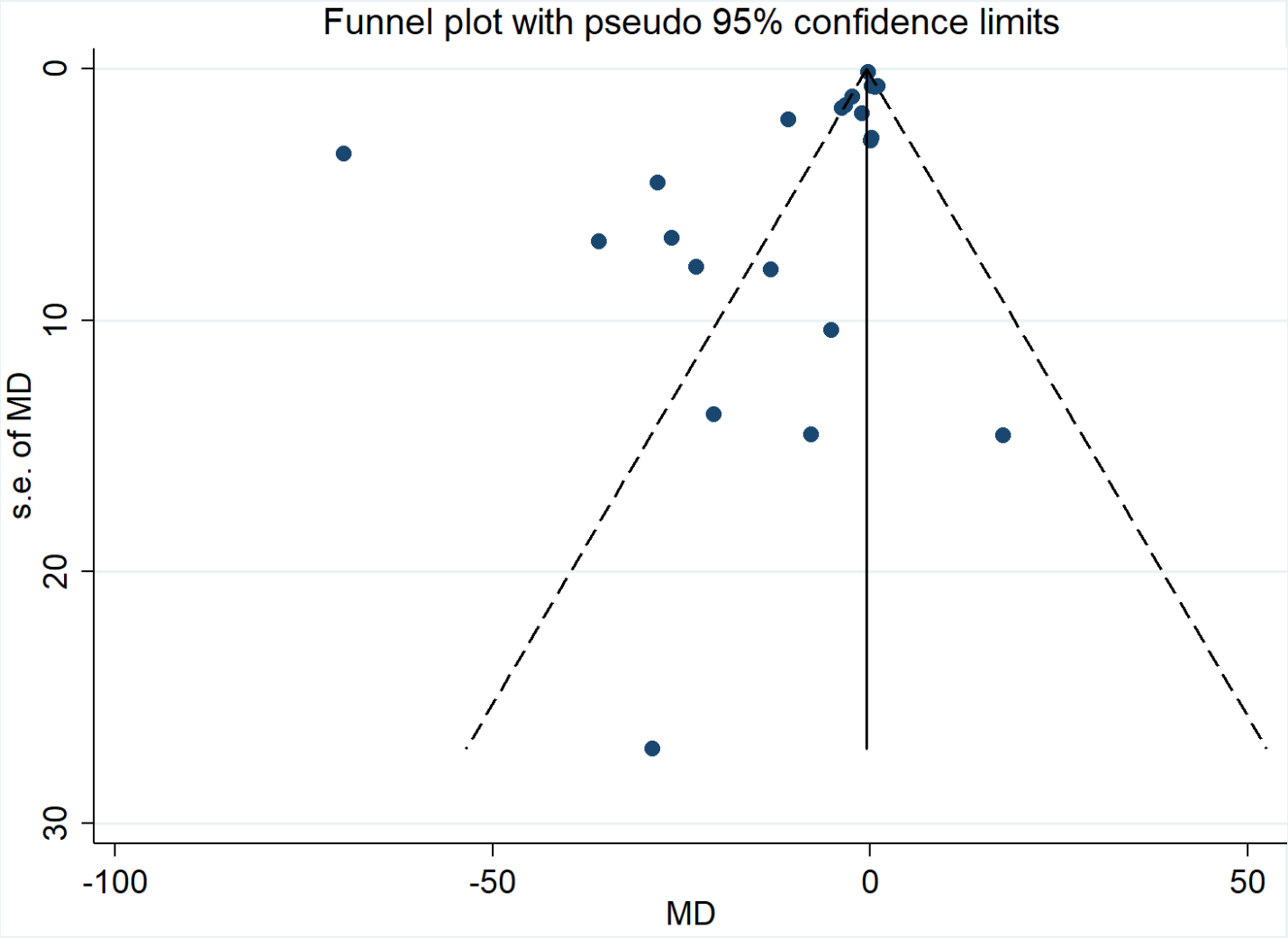


**Figure S1**. Funnel plot of FBG


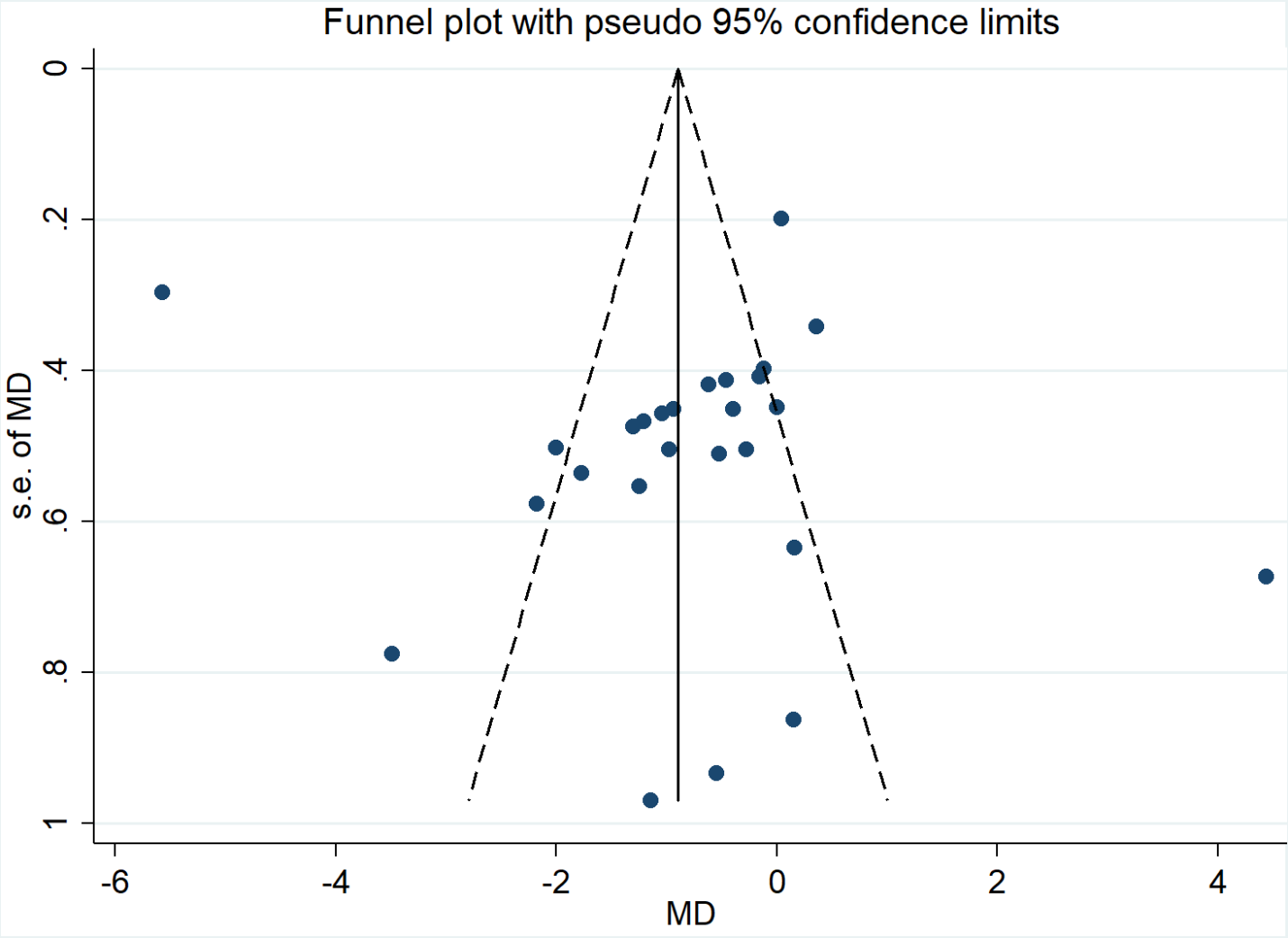


**Figure S2**. Funnel plot of PPG


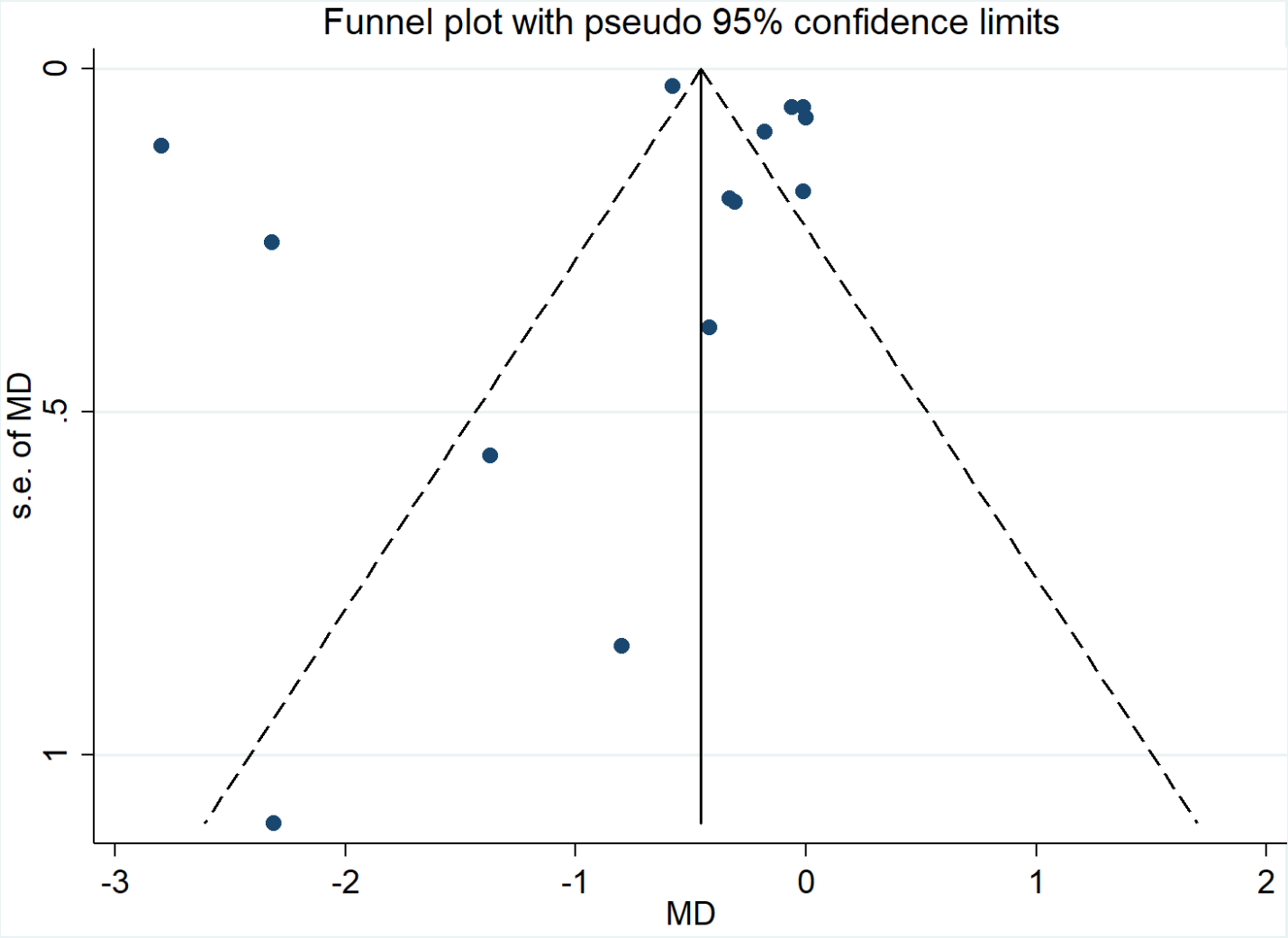


**Figure S3**. Funnel plot of HbA1C


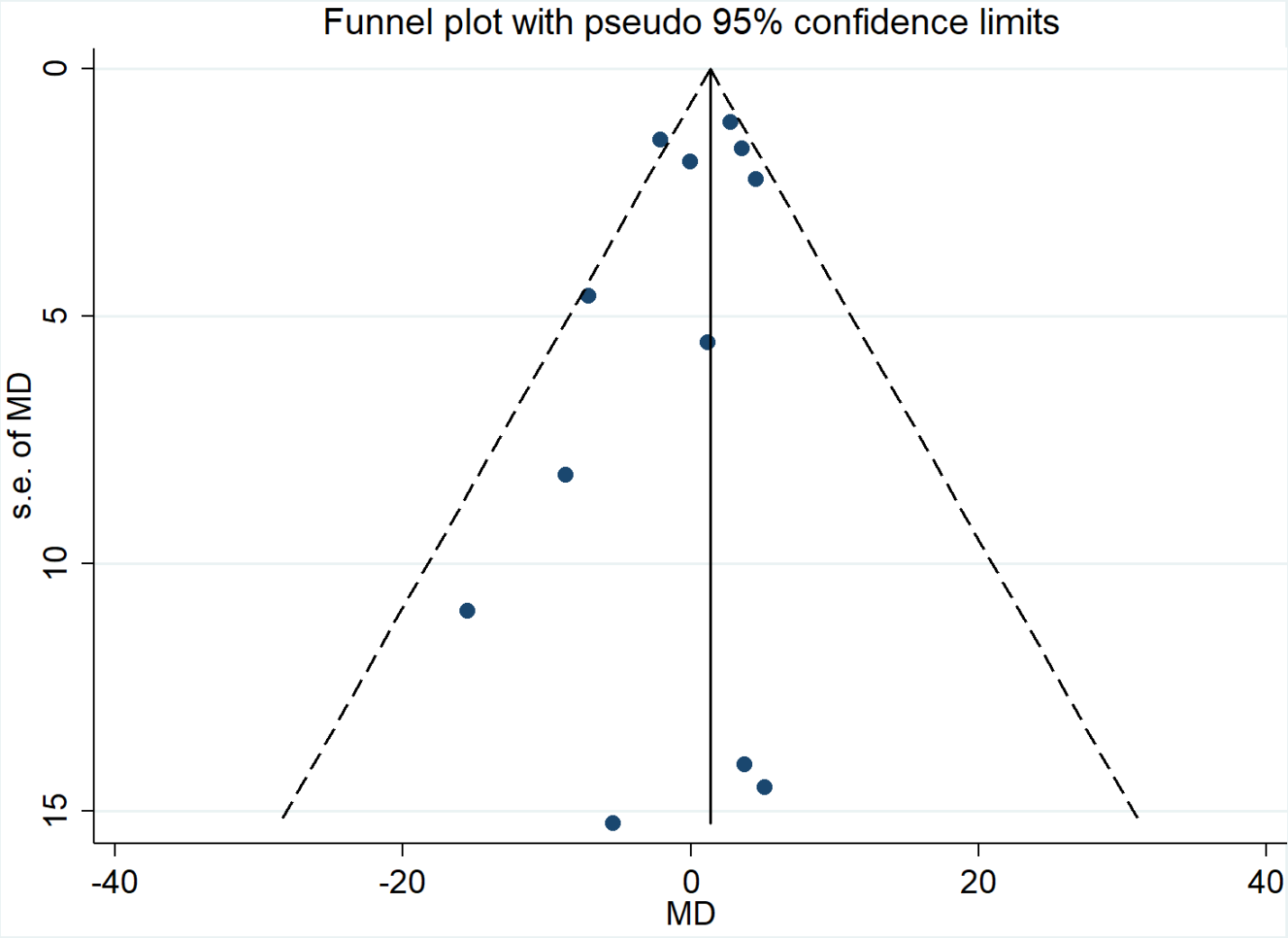


**Figure S4**. Funnel plot of FPI


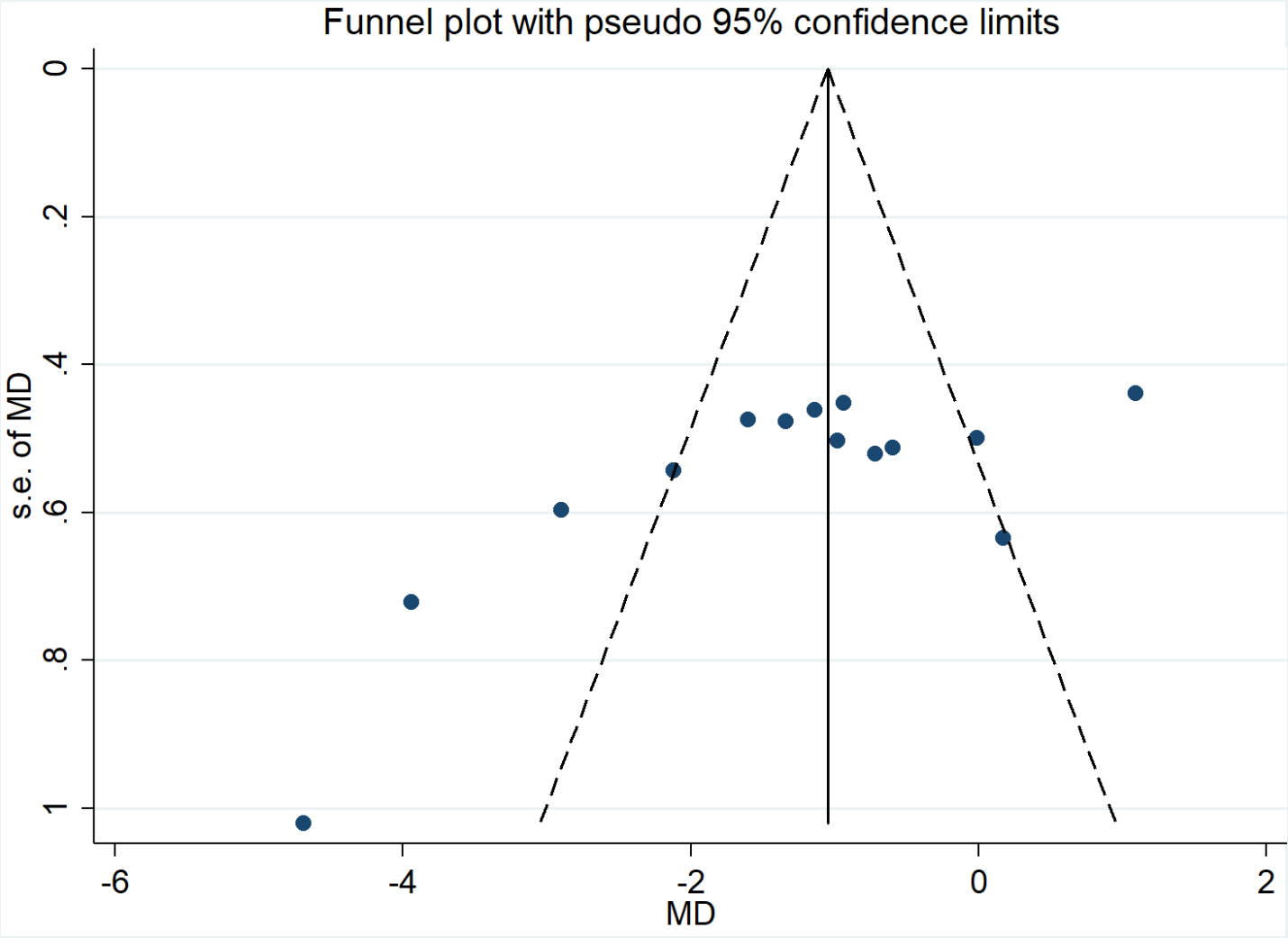


**Figure S5**. Funnel plot of PPI


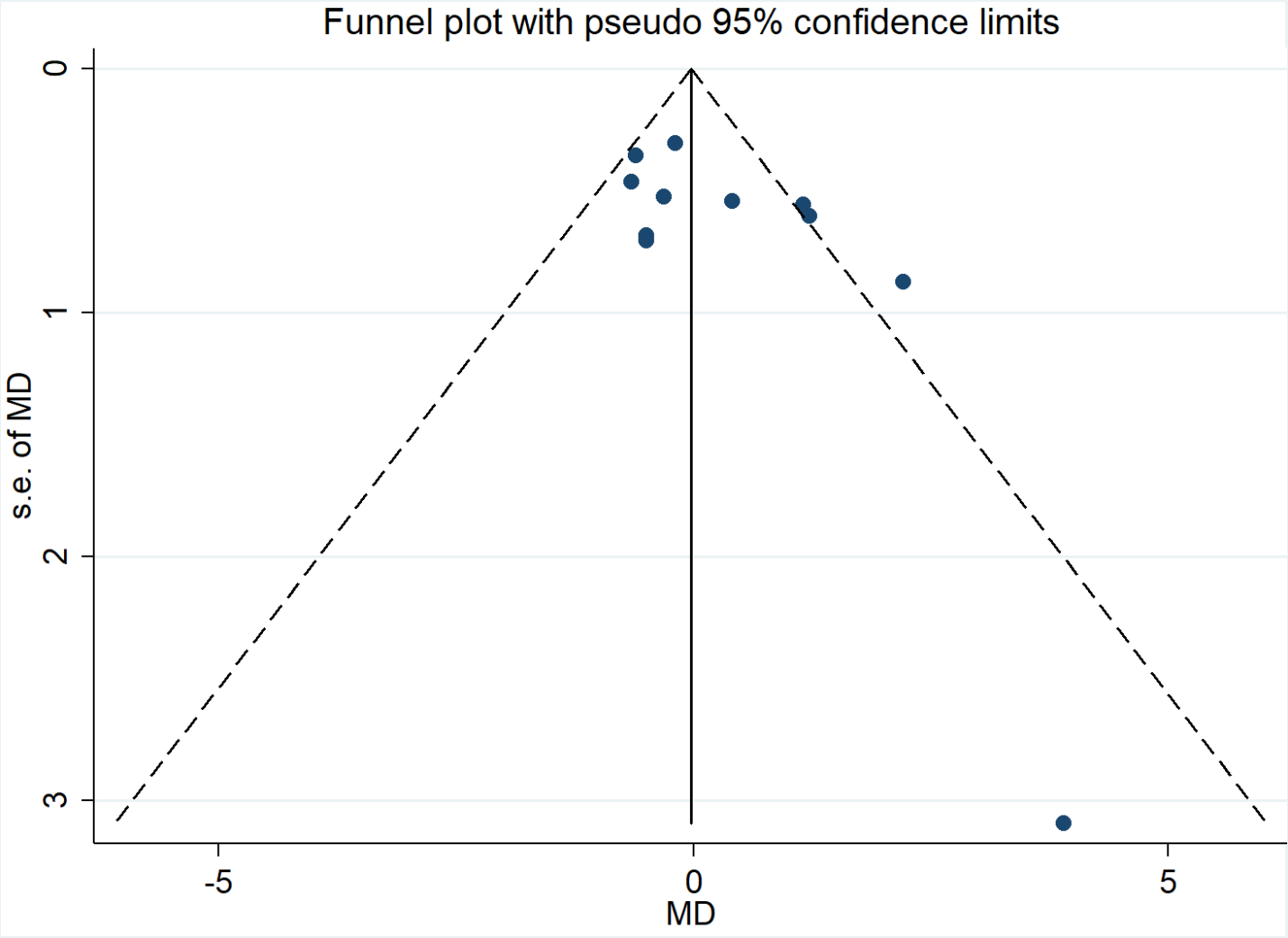


**Figure S6**. Funnel plot of HOMA-IR


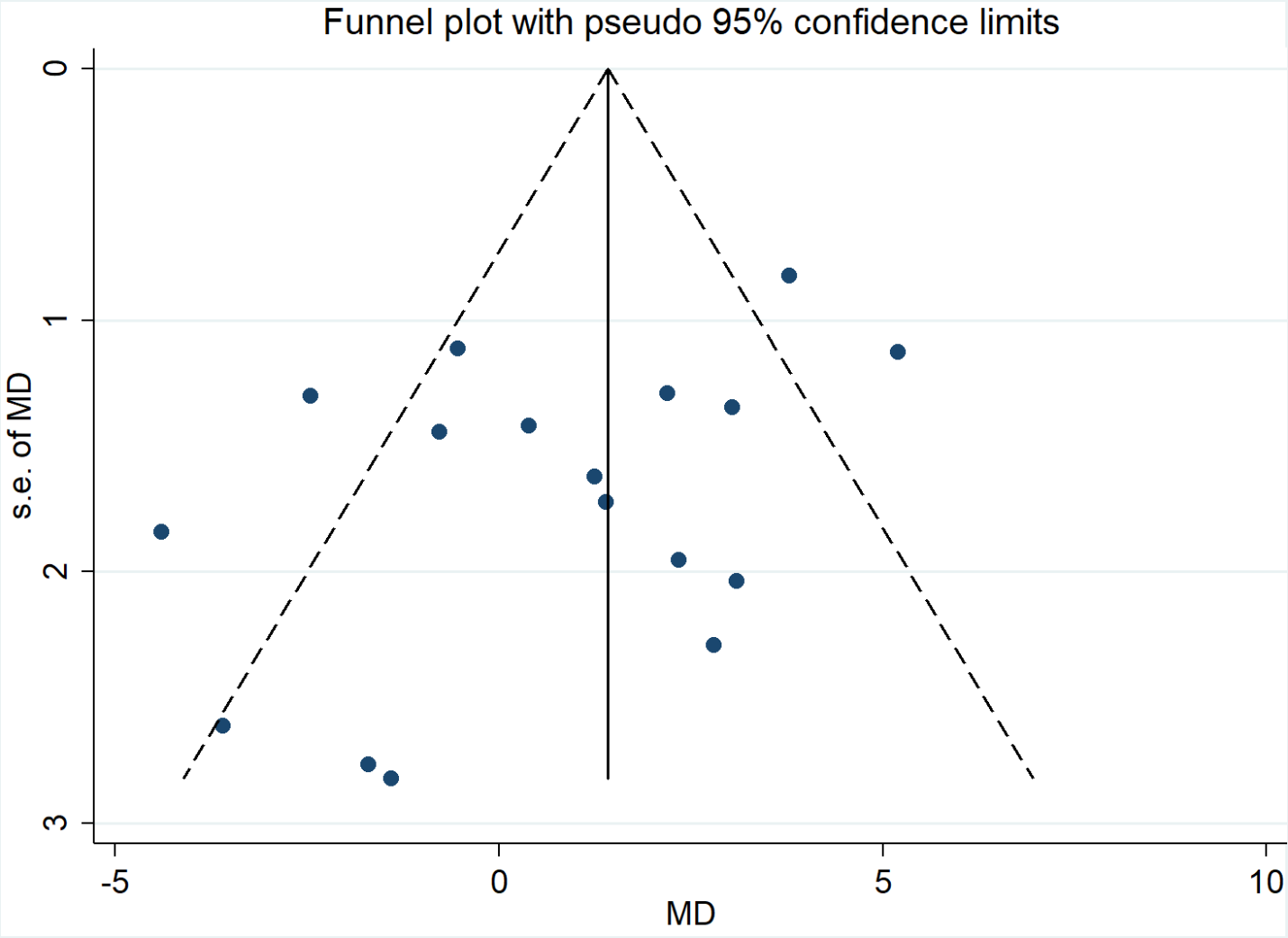


**Figure S7**. Funnel plot of HDL


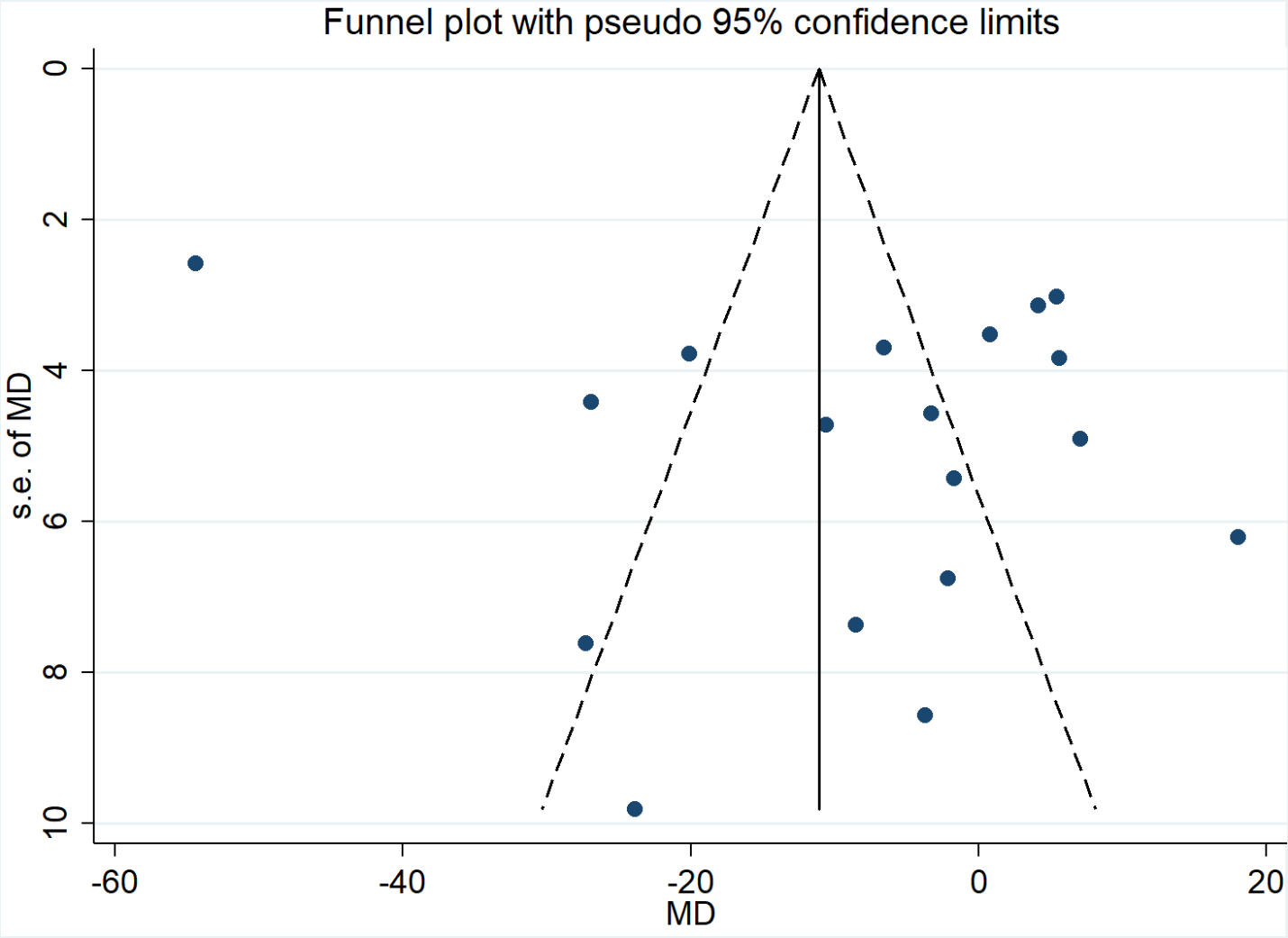


**Figure S8**. Funnel plot of LDL


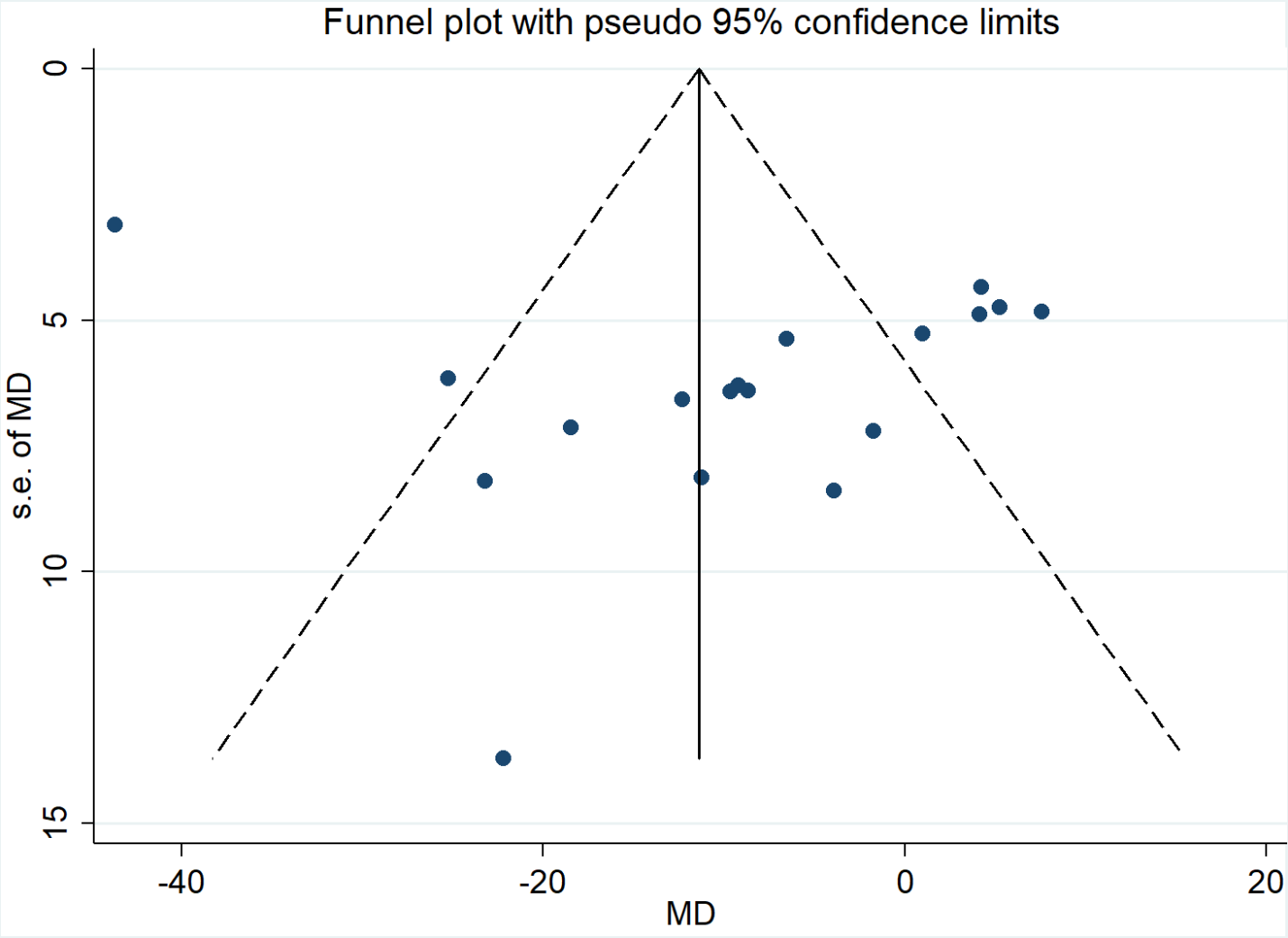


**Figure S9**. Funnel plot of TC


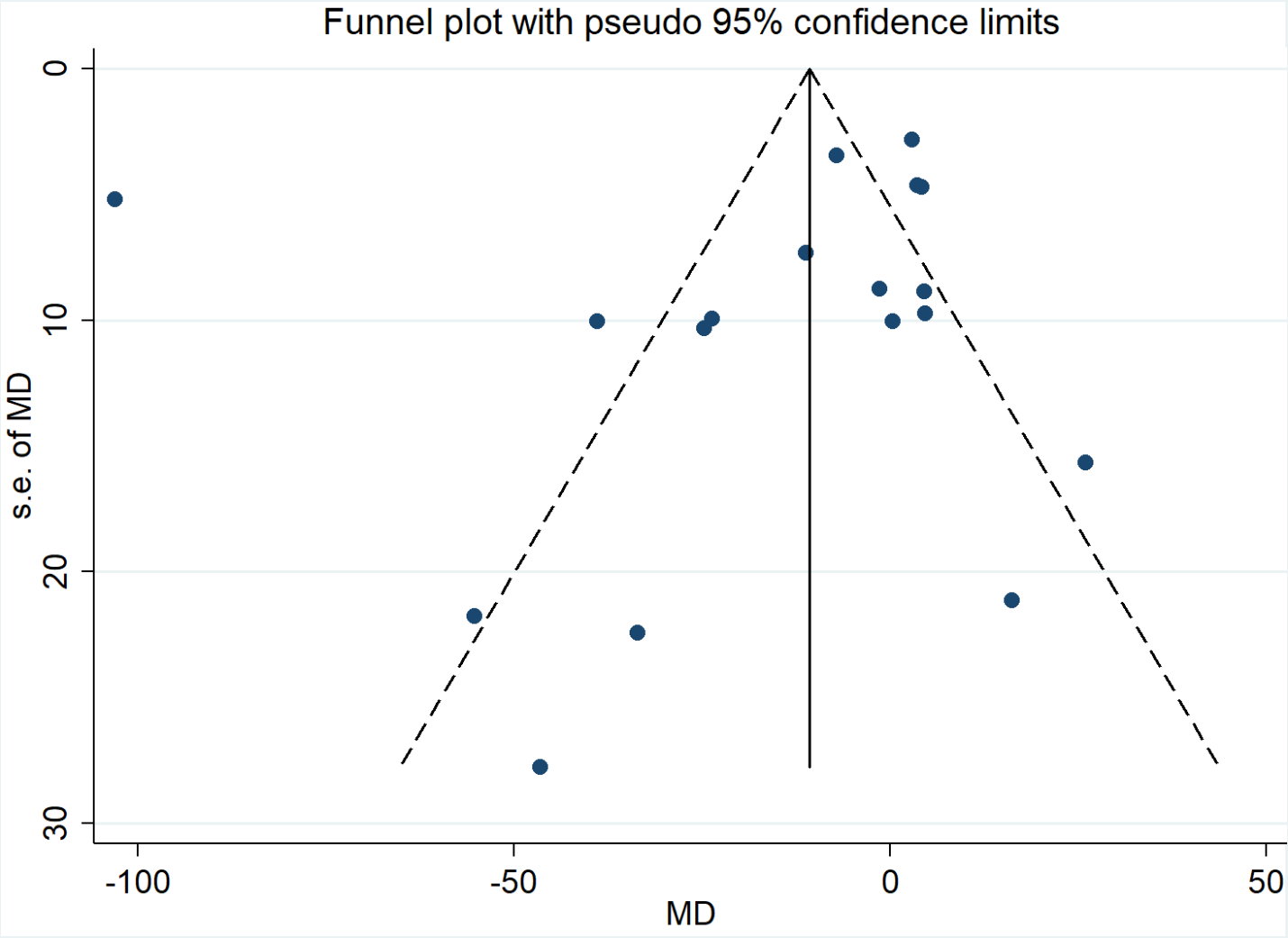


**Figure S10**. Funnel plot of TG


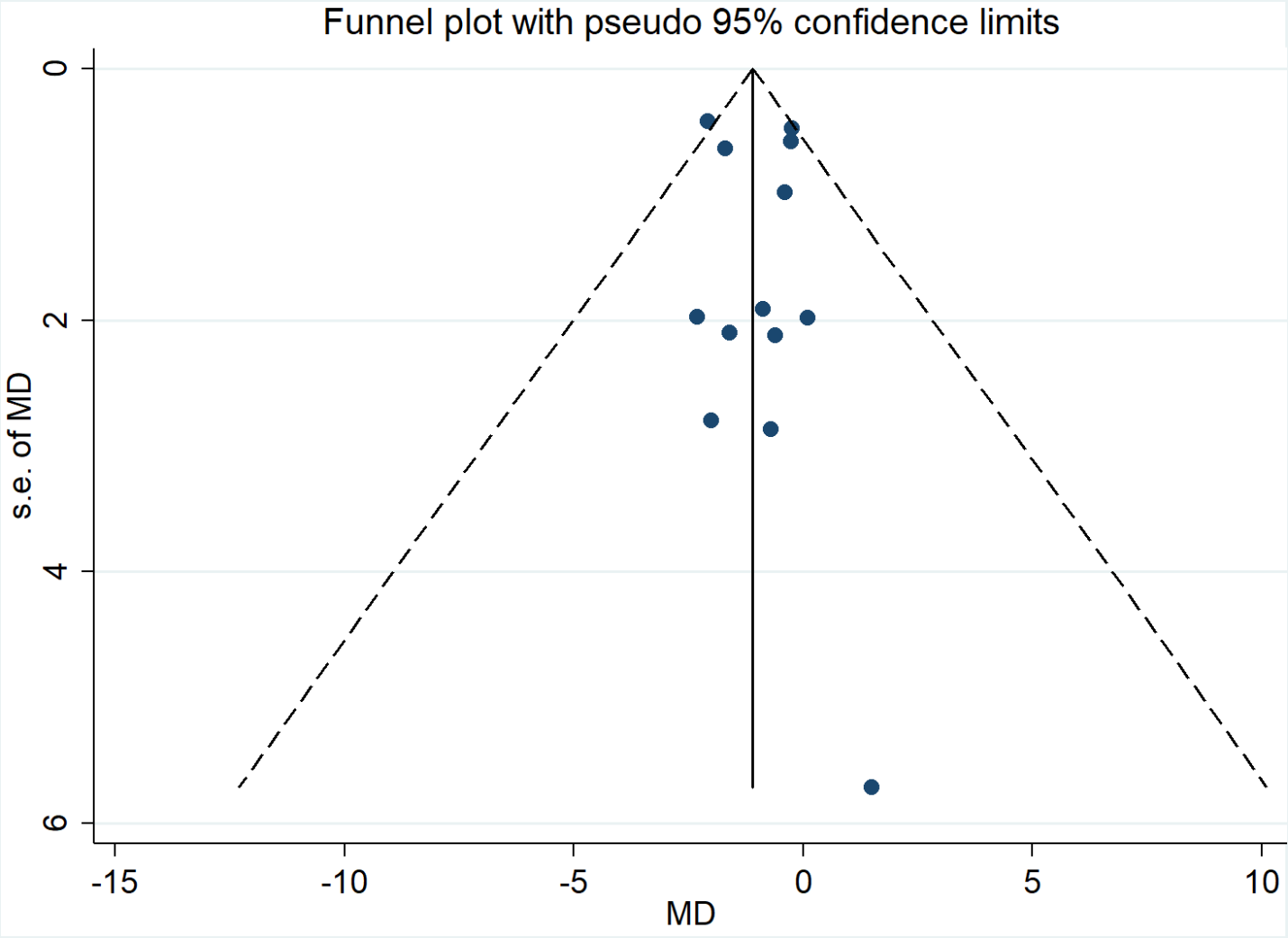


**Figure S11**. Funnel plot of weight


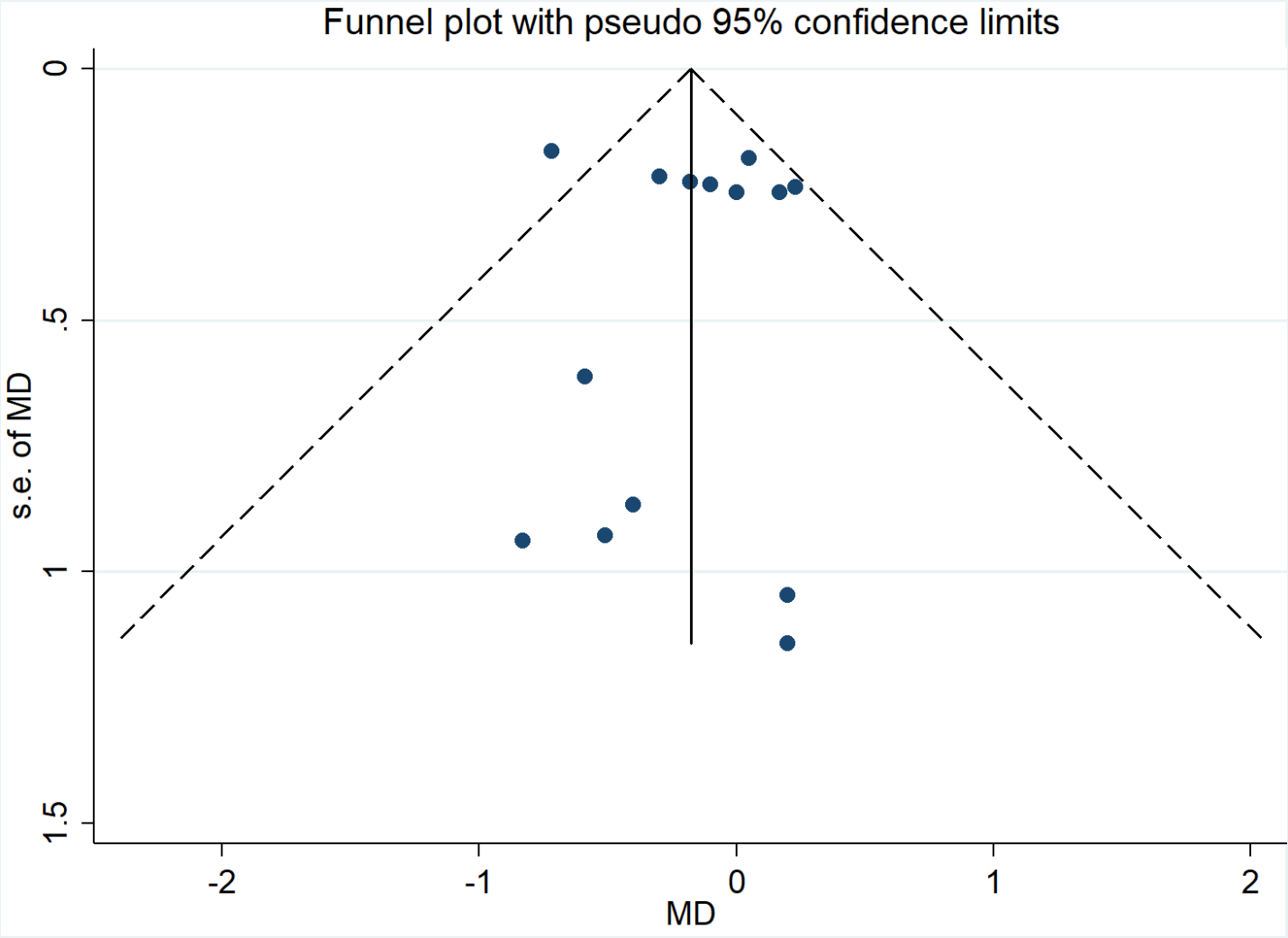


**Figure S12**. Funnel plot of BMI


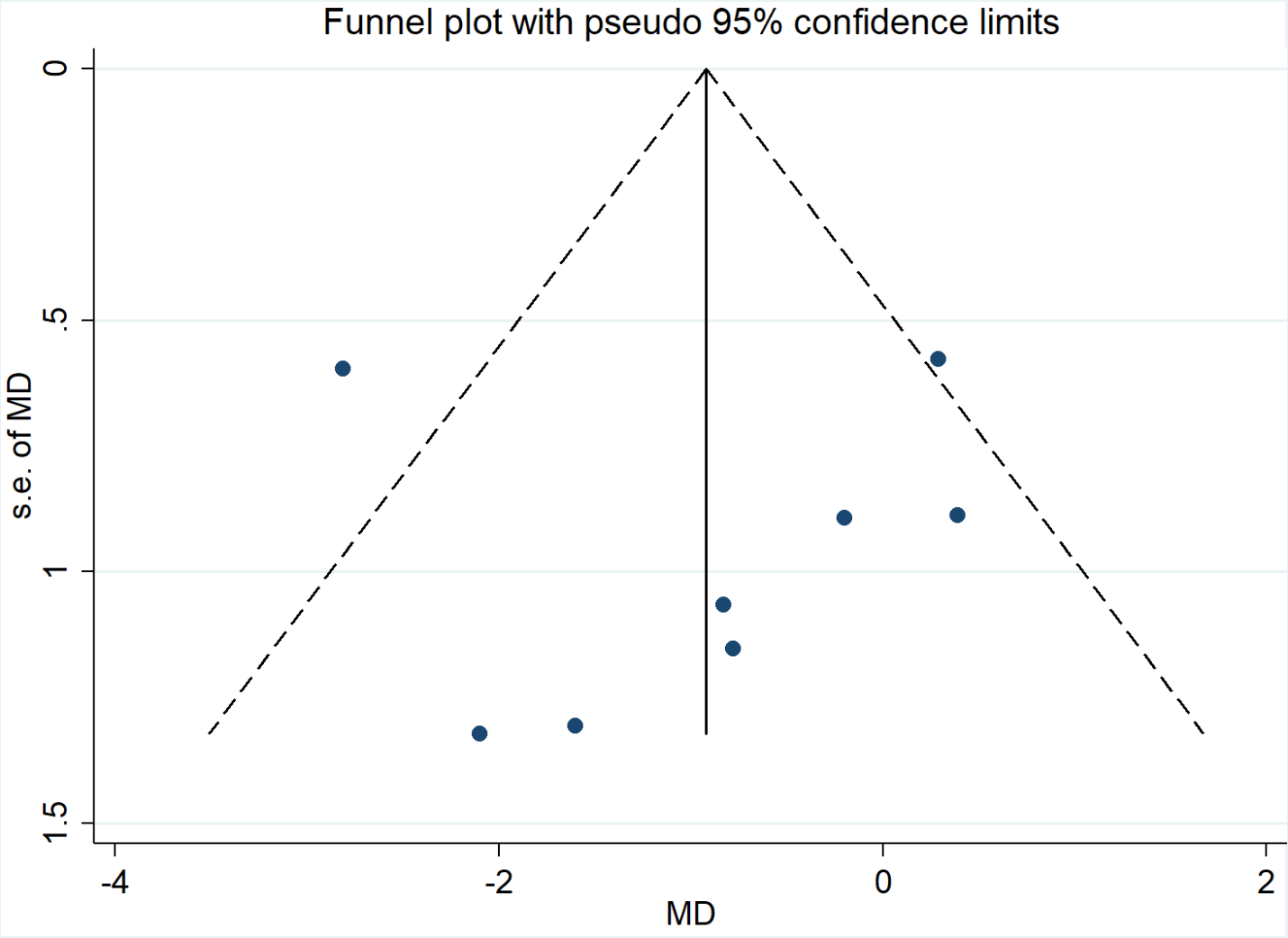


**Figure S13**. Funnel plot of WC


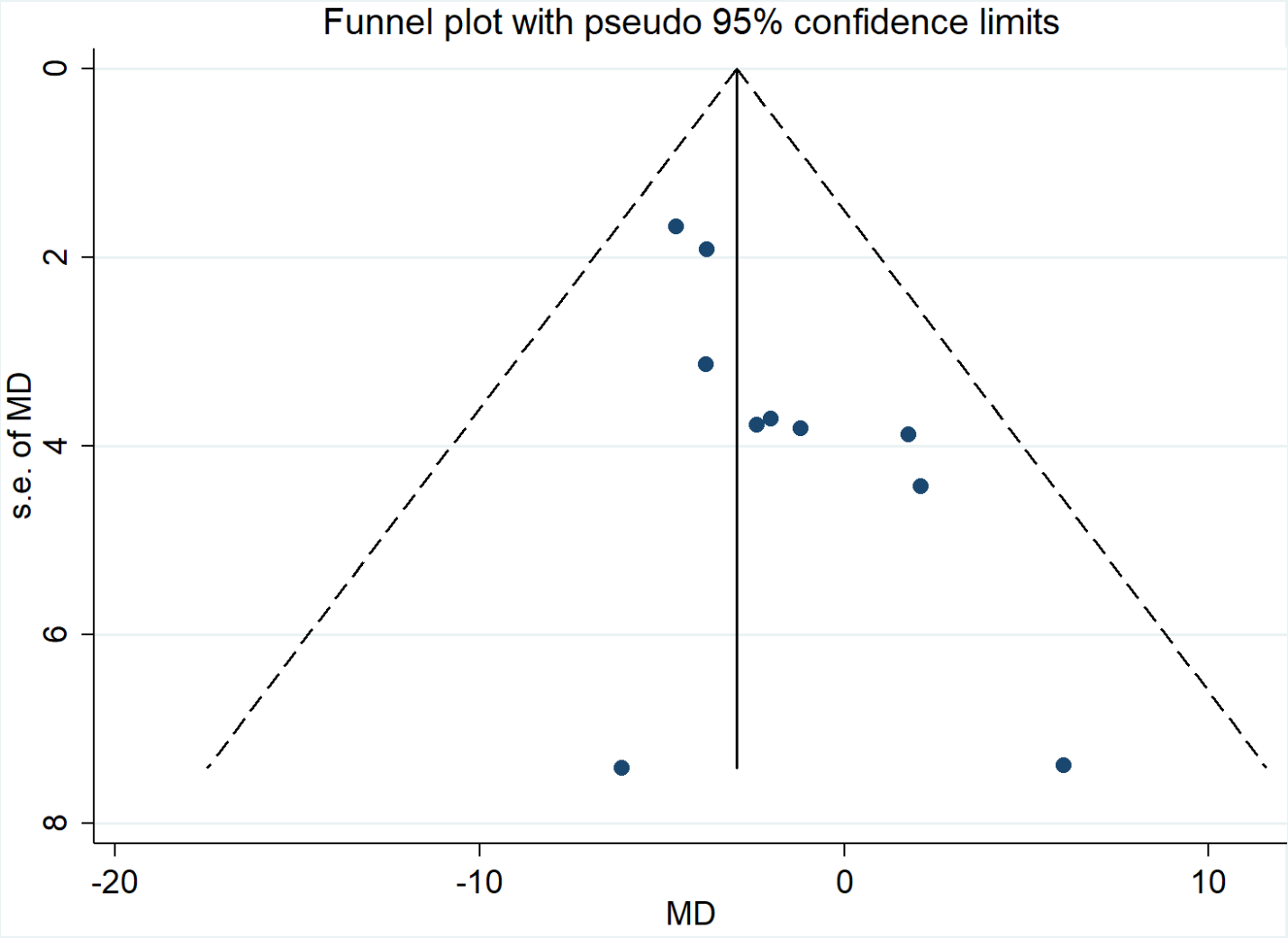


**Figure S14**. Funnel plot of SBP


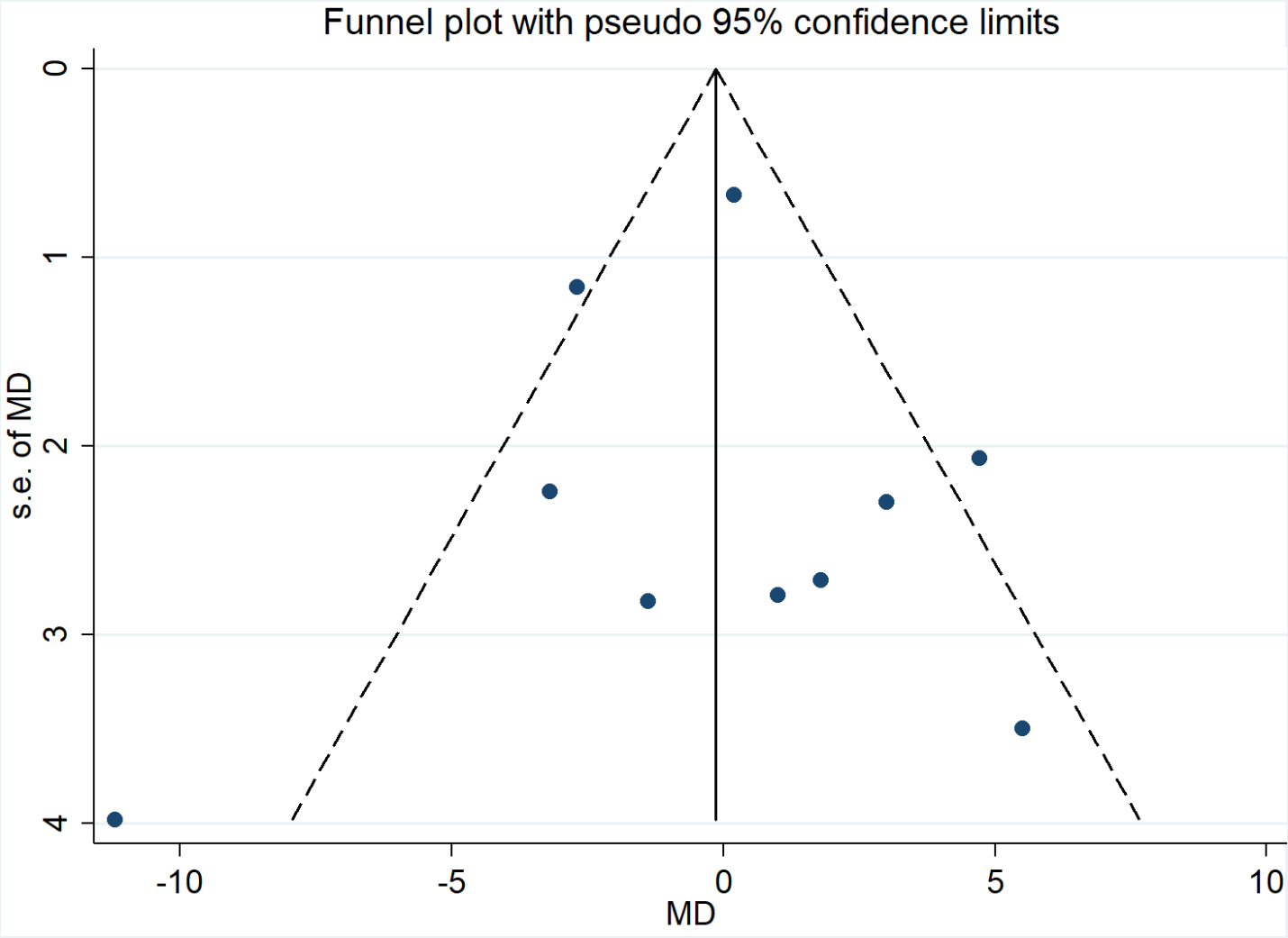


**Figure S15**. Funnel plot of DBP

**References:**

1. Chan M, Larsen N, Baxter H, Jespersen L, Ekinci EI, Howell K. The impact of botanical fermented foods on metabolic syndrome and type 2 diabetes: a systematic review of randomised controlled trials. Nutrition Research Reviews. 2023.

2. Hasan FO, Hamilton KP, Angadi SS, Kranz S. Effects of Vinegar/Acetic Acid Intake on Appetite Measures and Energy Consumption: Systematic Review. Translational Journal of the American College of Sports Medicine. 2022;7(3).

3. Launholt TL, Kristiansen CB, Hjorth P. Safety and side effects of apple vinegar intake and its effect on metabolic parameters and body weight: a systematic review. European Journal of Nutrition. 2020;59(6):2273-89.

4. Wang T, Xu TC, Xie JW, Li D. Effect of vinegar on glycemia: A meta-analysis. Chinese Journal of Clinical Nutrition. 2009;17(3):144-7.

5. Weber I, Woolhiser E, Keime N, Wasvary M, Adelman MJ, Sivesind TE, et al. Clinical Efficacy of Nutritional Supplements in Atopic Dermatitis: Systematic Review. JMIR Dermatol. 2023;6:e40857.

6. Astbury NM. Interventions to improve glycaemic control in people living with, and at risk of developing type 2 diabetes. Diabetes, Obesity and Metabolism. 2024;26(S4):39-49.

7. Brown LR, Sousa MS, Yule MS, Baracos VE, McMillan DC, Arends J, et al. Body weight and composition endpoints in cancer cachexia clinical trials: Systematic Review 4 of the cachexia endpoints series. Journal of Cachexia, Sarcopenia and Muscle. 2024;15(3):816-52.

8. DeSalvo JC, Skiba MB, Howe CL, Haiber KE, Funk JL. Natural Product Dietary Supplement Use by Individuals With Rheumatoid Arthritis: A Scoping Review. Arthritis Care Res (Hoboken). 2019;71(6):787-97.

9. Feng Y, Pan M, Li R, He W, Chen Y, Xu S, et al. Recent developments and new directions in the use of natural products for the treatment of inflammatory bowel disease. Phytomedicine. 2024;132.

10. Gill PA, van Zelm MC, Muir JG, Gibson PR. Review article: short chain fatty acids as potential therapeutic agents in human gastrointestinal and inflammatory disorders. Alimentary Pharmacology and Therapeutics. 2018;48(1):15-34.

11. Harrison F, Blower A, de Wolf C, Connelly E. Sweet and sour synergy: exploring the antibacterial and antibiofilm activity of acetic acid and vinegar combined witmedical-grade honeys. Microbiology (United Kingdom). 2023;169(7).

12. Liu W, Zhang Y, Zheng M, Ye Y, Shi M, Wang X, et al. Polysaccharides in Medicinal and Food Homologous Plants regulate intestinal flora to improve type 2 diabetes: Systematic review. Phytomedicine. 2024;134.

13. Marmitt DJ, Shahrajabian MH, Goettert MI, Rempel C. Clinical trials with plants in diabetes mellitus therapy: a systematic review. Expert Review of Clinical Pharmacology. 2021;14(6):735-47.

14. Medina-Vera I, Gómez-de-Regil L, Gutiérrez-Solis AL, Lugo R, Guevara-Cruz M, Pedraza-Chaverri J, et al. Dietary Strategies by Foods with Antioxidant Effect on Nutritional Management of Dyslipidemias: A Systematic Review. Antioxidants. 2021;10(2).

15. Neffe-Skocinska K, Karbowiak M, Kolozyn-Krajewska D, Zielinska D. Polyphenol and antioxidant properties of food obtained by the activity of acetic acid bacteria (AAB)-A systematic review. Journal of Functional Foods. 2023;107.

16. Sadia S, Tariq A, Shaheen S, Malik K, khan F, Ahmad M, et al. Ethnopharmacological profile of anti-arthritic plants of Asia-a systematic review. Journal of Herbal Medicine. 2018;13:8-25.

17. Shahrajabian MH, Marmitt DJ, Cheng Q, Sun W. Natural Antioxidants of the Underutilized and Neglected Plant Species of Asia and South America. Letters in Drug Design and Discovery. 2023;20(10):1512-37.

18. Xavier L, Reis TCG, Martins ASD, Santos JCD, Bueno NB, Goulart MOF, et al. Antioxidant Therapy in Inflammatory Bowel Diseases: How Far Have We Come and How Close Are We? Antioxidants. 2024;13(11).

19. Zhang Y, Zeng M, Zhang XL, Yu Q, Zeng WY, Yu B, et al. Does an apple a day keep away diseases? Evidence and mechanism of action. Food Science & Nutrition. 2023;11(9):4926-47.

20. Tehrani SD, Keshani M, Rouhani MH, Moallem SA, Bagherniya M, Sahebkar A. The effects of apple cider vinegar on cardiometabolic risk factors: A systematic review and meta-analysis of clinical trials. Curr Med Chem. 2023.

21. Sohouli MH, Kutbi E, Al Masri MK, Dadhkhah H, Fatahi S, Santos HO, et al. Effects of vinegar consumption on cardiometabolic risk factors: A systematic review and meta-analysis of randomized controlled trials. European Journal of Integrative Medicine. 2022;55.

22. Shahinfar H, Amini MR, Payandeh N, Torabynasab K, Pourreza S, Jazayeri S. Dose-dependent effect of vinegar on blood pressure: A GRADE-assessed systematic review and meta-analysis of randomized controlled trials. Complementary Therapies in Medicine. 2022;71.

23. Fakhri M, Fatahian A, Yousefi SS, Moosazadeh M, Azadbakht M. The effect of natural products use on blood pressure in Iran: Systematic review and meta-analysis. Journal of Nursing and Midwifery Sciences. 2022;9(2):152-65.

24. Cheng LJ, Jiang Y, Wu VX, Wang WR. A systematic review and meta-analysis: Vinegar consumption on glycaemic control in adults with type 2 diabetes mellitus. Journal of Advanced Nursing. 2020;76(2):459-74.

25. Hadi A, Pourmasoumi M, Najafgholizadeh A, Clark CCT, Esmaillzadeh A. The effect of apple cider vinegar on lipid profiles and glycemic parameters: a systematic review and meta-analysis of randomized clinical trials. Bmc Complementary Medicine and Therapies. 2021;21(1).

26. Siddiqui FJ, Assam PN, de Souza NN, Sultana R, Dalan R, Chan ESY. Diabetes Control: Is Vinegar a Promising Candidate to Help Achieve Targets? Journal of Evidence-Based Integrative Medicine. 2018;23.

27. Shishehbor F, Mansoori A, Shirani F. Vinegar consumption can attenuate postprandial glucose and insulin responses; a systematic review and meta-analysis of clinical trials. Diabetes Research and Clinical Practice. 2017;127:1-9.

28. Arjmandfard D, Behzadi M, Sohrabi Z, Mohammadi Sartang M. Effects of apple cider vinegar on glycemic control and insulin sensitivity in patients with type 2 diabetes: A GRADE-assessed systematic review and dose-response meta-analysis of controlled clinical trials. Front Nutr. 2025;12:1528383.

29. Valdes DS, So D, Gill PA, Kellow NJ. Effect of Dietary Acetic Acid Supplementation on Plasma Glucose, Lipid Profiles, and Body Mass Index in Human Adults: A Systematic Review and Meta-analysis. Journal of the Academy of Nutrition and Dietetics. 2021;121(5):895-914.
